# Integration of personalised ultrasensitive ctDNA monitoring of patients with metastatic breast cancer to reduce imaging requirements

**DOI:** 10.1002/ijc.35292

**Published:** 2024-12-18

**Authors:** Pia Mouhanna, Anders Ståhlberg, Daniel Andersson, Ahmed Albu‐Kareem, Ellinor Elinder, Olle Eriksson, Amy Kavanagh, Anikó Kovács, Karolina F. Larsson, Barbro Linderholm, Monika Uminska, Tobias Österlund, Sacha J. Howell, Maria Ekholm

**Affiliations:** ^1^ Sahlgrenska Center for Cancer Research, Department of Laboratory Medicine, Institute of Biomedicine, Sahlgrenska Academy University of Gothenburg Gothenburg Sweden; ^2^ Department of Oncology Ryhov County Hospital Jönköping Sweden; ^3^ Department of Clinical Genetics and Genomics Sahlgrenska University Hospital Gothenburg Sweden; ^4^ Wallenberg Centre for Molecular and Translational Medicine University of Gothenburg Gothenburg Sweden; ^5^ Department of Oncology Linköping University Hospital Linköping Sweden; ^6^ Department of Oncology Södersjukhuset Stockholm Sweden; ^7^ Futurum – The Academy for Health and Care Jönköping County Sweden; ^8^ Department of Medical Oncology The Christie NHS Foundation Trust Manchester UK; ^9^ Department of Clinical Pathology Sahlgrenska University Hospital Gothenburg Sweden; ^10^ Sahlgrenska Center for Cancer Research, Department of Oncology, Institute of Clinical Sciences, Sahlgrenska Academy University of Gothenburg Gothenburg Sweden; ^11^ Department of Oncology Sahlgrenska University Hospital Gothenburg Sweden; ^12^ Department of Oncology Kalmar County Hospital Kalmar Sweden; ^13^ Division of Cancer Sciences, Faculty of Biology, Medicine and Health The University of Manchester Manchester UK; ^14^ Department of Biomedical and Clinical Sciences, Division of Oncology Linköping University Linköping Sweden

**Keywords:** breast cancer, ctDNA, imaging, liquid biopsy, precision medicine

## Abstract

Circulating tumour DNA (ctDNA) is an emerging biomarker for monitoring cancers. The personalised disease monitoring in metastatic breast cancer (PDM‐MBC) study is an ongoing study instigated to evaluate ctDNA as a biomarker to individualise imaging requirements in patients with MBC. Patients receiving first‐line endocrine therapy (aromatase inhibitor + cyclin‐dependent kinase 4/6 inhibitor) had plasma samples collected pre‐treatment, weeks 2 and 4, and concurrently with imaging until progressive disease (PD). Here, we apply an experimental analytical workflow for ultrasensitive ctDNA analysis, utilising personalised ctDNA panels designed from mutations identified in tumour tissue, and present results for 24 patients. Twenty patients (83%) had detectable ctDNA pre‐treatment. The median progression‐free survival was 25.6 months, and 13 patients experienced PD, with rising ctDNA detected at or prior to PD in 12 patients (92%). If imaging had been omitted until the detection of rising ctDNA for at least one mutation, 68% (*n* = 71) of the scans performed amongst ctDNA‐positive patients would have been avoided. Our results demonstrate that integration of personalised ctDNA monitoring of patients with MBC has potential to substantially reduce the imaging needs in patients showing ctDNA response to treatment.

## Graphical abstract

What's new?

ctDNA is a valuable tool for cancer detection, owing to its potential to facilitate early disease detection and to inform treatment decisions for personalised medicine. The present report describes the PDM‐MBC study, in which the potential use of ctDNA analysis to guide imaging needs in MBC patients is investigated. Among 24 patients, 83% had detectable ctDNA prior to MBC treatment. For most patients, rising levels of ctDNA predicted PD. Moreover, among ctDNA‐positive patients, approximately two‐thirds of all scans performed were rendered unnecessary through ctDNA analysis.

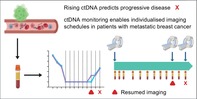

## 1 INTRODUCTION

International consensus guidelines recommend regular imaging of metastatic lesions every 2–4 months to assess treatment response in patients with metastatic breast cancer (MBC).^1^ Imaging procedures can, however, pose challenges for patients, including physical and psychological morbidity, the latter often reported in the run‐up to imaging and whilst waiting for results.^2^ Imaging also imposes substantial financial and workforce strain on healthcare systems. Moreover, response assessment through imaging can be challenging for non‐measurable metastases in bone, pleura, and peritoneum,^3^, ^4^ and is prone to inter‐observer variability.^5^ Biomarkers of response and progressive disease (PD) that can safely reduce the number of regular imaging time points are desirable. Integration of circulating tumour DNA (ctDNA) with imaging response assessment has recently been highlighted as a future application of ctDNA in MBC.^6^, ^7^

The Personalised Disease Monitoring in MBC (PDM‐MBC) study is an ongoing prospective biomarker study, investigating the potential use of personalised ctDNA analysis to guide imaging needs in patients treated for MBC. We describe here our experimental analytical workflow and report data for the first 24 patients for whom tumour tissue was sequenced for personalised ctDNA analysis.

## 2 METHODS

### 2.1 Study design

Patients with MBC, eligible for first line endocrine therapy with an aromatase inhibitor + cyclin‐dependent kinase 4/6 inhibitor (CDK4/6i), were recruited across six sites in Sweden and England between June 2019 and March 2023. Patients without PD will be followed until May 2026. Computed tomography (CT) ± magnetic resonance imaging (MRI) was performed every 3–4 months. PD was defined as either radiological progression by response evaluation criteria in solid tumours (RECIST) 1.1,^8^ or clinical progression in the opinion of the treating clinician. For example, this included marked deterioration in liver function tests or progression of metastases not well visualised on imaging, such as those in skin or soft tissue. Blood samples for ctDNA analysis were collected pre‐treatment, at cycle 1 day 15 (C1d15) and cycle 2 day 1 (C2d1), and thereafter concurrently with imaging until PD, while on CDK4/6i.

### 2.2 Personalised ctDNA analysis

**FIGURE 1:**
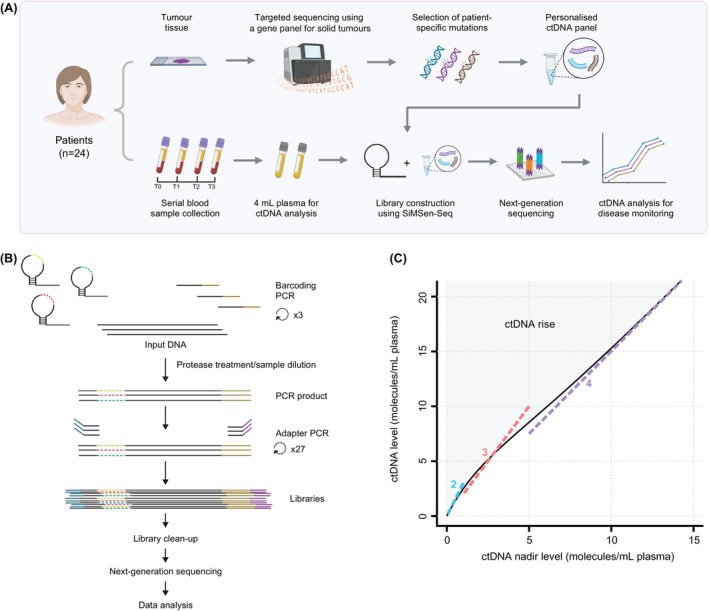
Personalised ctDNA analysis using SiMSen‐Seq. (A) Overview of the schematic workflow for personalised ctDNA analysis. To identify patient‐specific mutations, tumour tissue DNA was sequenced using a gene panel for solid tumours. Personalised ctDNA panels were designed and validated. Cell‐free DNA was extracted from serially collected blood plasma and assessed for presence of ctDNA using SiMSen‐Seq. (Created in BioRender. Ståhlberg (2024) https://BioRender.com/b07j471.) (B) Overview of the SiMSen‐Seq workflow. In the barcoding PCR, unique molecular identifiers, protected in a stem‐loop structure, are attached to individual target DNA molecules. In the adapter PCR, barcoded target DNA is amplified with sequencing adapters. Final libraries are purified and sequenced. The unique molecular identifiers are bioinformatically used to minimise sequencing errors. (Created in BioRender. Ståhlberg (2024) https://BioRender.com/q78i200.) (C) To detect rising ctDNA, levels (molecules/mL) were compared to ctDNA_nadir_, defined as the lowest previously observed level (disregarding the C1d15 time point). Any of the following criteria corresponded to a ctDNA rise: (1) reappearance of ctDNA if ctDNAnadir was below the threshold for detection, or detection of ctDNA if previously undetected; (2) >3‐fold increase in ctDNA level if ctDNA_nadir_ ≤1 molecule/mL; (3) >2‐fold increase if ctDNA_nadir_ >1–5 molecules/mL; (4) >1.5‐fold increase if ctDNA_nadir_ >5 molecules/mL. Criteria 2–4 are depicted as segments and the black line represents the limit function, expressed as ctDNA_nadir_ × (1.5 + 1.47 × e^−0.39×ctDNAnadir^) that was devised to facilitate a smooth transition between these criteria (see Supplementary Methods). Rising ctDNA was considered to exist if the ctDNA level for ≥1 mutation exceeded the value of the limit function. Abbreviation: ctDNA, circulating tumour DNA.

Blood samples were collected into EDTA tubes and cell‐free DNA was extracted from plasma using either the QIAsymphony DSP Circulating DNA Kit or manually using the QIAmp Circulating Nucleic Acid Kit (both Qiagen). We used a tumour‐informed, personalised approach to analyse ctDNA, and the workflow is outlined in Figure 1A. Mutations were identified through sequencing of tumour tissue DNA, utilising a hotspot panel targeting 544 genes.^9^ We designed and validated personalised ctDNA panels for each patient, which were used to analyse ctDNA. We applied simple multiplexed PCR‐based barcoding of DNA for sensitive mutation detection using sequencing (SiMSen‐Seq) to quantify ctDNA levels in plasma as previously described,^10^ allowing detection of single ctDNA molecules (Figure 1B). SiMSen‐Seq libraries were sequenced on either a MiniSeq or NextSeq (both Illumina). Raw sequencing reads were bioinformatically processed using UMIErrorCorrect.^11^ Briefly, sequencing reads were collapsed into consensus reads, requiring ≥3 reads for each unique molecular identifier family. A single nucleotide mutation was considered true positive if detected in ≥2 consensus reads and with a mutant allele frequency (MAF) of ≥0.1%, while insertions and deletions only required one consensus read. The criteria for ctDNA rise are shown in Figure 1C and further described in Supplementary Methods and Supplementary Table 4A, B, and C. The sequencing coverage and quality statistics for each sample are summarised in Supplementary Table 5.

### 2.3 Personalised imaging schedules

Scans performed prior to or without rising ctDNA were classified as ‘unnecessary’, whereas scans performed at or after the first ctDNA rise, or in the absence of a blood draw, were classified as ‘necessary’. All scans performed after the first ctDNA rise were classified as ‘necessary’, whether or not ctDNA levels decreased or became undetectable thereafter. One scan was counted per time point, although some patients underwent both CT and MRI per protocol.

Detailed information on study procedures and methodology is provided in Supplementary Methods.

## 3 RESULTS

### 3.1 Patient characteristics and clinical outcome

Patient and disease characteristics, along with clinical outcomes, are presented in Table 1, with more detailed information provided in Supplementary Table 1. Thirteen patients (54%) developed PD, and 9 patients (38%) had died by the data cut‐off date. The median progression‐free survival (PFS) was 25.6 months (95% confidence interval [CI]: 6.5–44.7).

**TABLE 1:** Patient and disease characteristics and clinical outcome.

	All patients *n* = 24 (100)	ctDNA‐positive patients *n* = 20 (83)	ctDNA‐negative patients *n* = 4 (17)	*p*‐value
Median age, years (range)	55 (28–84)	56 (28–84)	55 (50–57)	1.0^d^
Performance status^a^				
0	15 (63)	12 (60)	3 (75)	1.0^e^
1	8 (33)	7 (35)	1 (25)	
2	1 (4)	1 (5)	0	
Disease				
De novo	10 (42)	8 (40)	2 (50)	1.0^e^
Recurrent	14 (58)	12 (60)	2 (50)	
TNM stage^b^				
III	1 (4)	1 (5)	0	1.0^e^
IV	23 (96)	19 (95)	4 (100)	
Metastatic lesions				
Bone‐predominant	4 (17)	4 (20)	0	0.80^e^
Visceral	12 (50)	10 (50)	2 (50)	
Other	8 (33)	6 (30)	2 (50)	
Measurable disease^c^				
Yes	17 (71)	14 (70)	3 (75)	1.0^e^
No	7 (29)	6 (30)	1 (25)	
Best response^a^				
Complete response	0	0	0	0.78^e^
Partial response	12 (50)	9 (45)	3 (75)	
Stable disease	9 (38)	8 (40)	1 (25)	
Progressive disease	3 (13)	3 (15)	0	
Median PFS, months (95% CI)	25.6 (6.5–44.7)	22.6 (5.7–39.6)	Not reached	0.062^f^
Progressive disease				
Yes	13 (54)	13 (65)	0	0.031^e^
No	11 (46)	7 (35)	4 (100)	
Death				
Yes	9 (38)	9 (45)	0	0.26^e^
No	15 (63)	11 (55)	4 (100)	
ctDNA rise at PD				
Yes	12 (92)	12 (92)	0	0.031^e^
No	1 (7.7)	1 (7.7)	0	

*Note*: Data are shown as number of patients with percentage within parenthesis unless otherwise noted.

Abbreviations: CI, confidence interval; ctDNA, circulating tumour DNA; ECOG, Eastern Cooperative Oncology Group; RECIST, Response Evaluation Criteria In Solid Tumours; PFS, progression‐free survival; PD, progressive disease.

^a^ According to ECOG.^12^

^b^ TMN classification for malignant tumours.^13^

^c^ According to RECIST 1.1.^8^

^d^ Mann Whitney U‐test.

^e^ Fisher's exact test.

^f^ Log‐rank test.

### 3.2 Personalised ctDNA analysis

Specific mutations were identified for each patient through targeted tumour tissue sequencing using either primary tumour (*n* = 14, 58%) or metastasis (*n* = 10, 42%). A median of 9 mutations (range 4–21) were suitable for ctDNA analysis and selected for ctDNA panel design (Supplementary Table 2). All personalised ctDNA panels were designed and validated with SiMSen‐Seq,^10^ an ultrasensitive sequencing method (Supplementary Figure 1). Detailed information about the ctDNA panels is reported in Supplementary Table 2. In total, 198 samples were analysed, with a median of 8 (range 4–14) samples per patient. Cell‐free DNA, range 2.75–842 ng, was extracted from a median of 2.80 mL (range 1.30–4.00) plasma per sample. SiMSen‐Seq libraries were then generated, purified, and sequenced, and data were successfully generated for all 198 plasma samples. Sequencing reads were collapsed into consensus reads that were used to estimate the number of ctDNA molecules per mL plasma and MAF.

### 3.3 The majority of patients displayed detectable ctDNA

**FIGURE 2:**
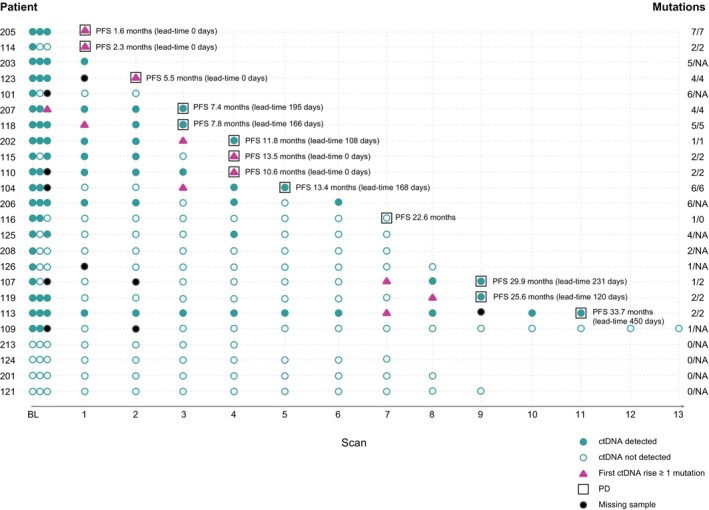
Summary of ctDNA data for all 24 patients in relation to clinical outcome. The first three time points correspond to samples collected pre‐treatment, after 2 weeks (C1d15), and after 4 weeks (C2d1). The ctDNA results and clinical outcome are denoted by symbols explained in the figure. Mutations refer to the number of detected mutations pre‐treatment and at PD. ctDNA levels and imaging results for the individual patients are provided in Supplementary Table 3 and Supplementary Figure 3. (Created in BioRender. Ståhlberg (2024) https://BioRender.com/q71y431.) Abbreviations: ctDNA, circulating tumour DNA; NA, not applicable; PD, progressive disease; PFS, progression‐free survival.

ctDNA results are summarised in Figure 2, with detailed information provided in Supplementary Table 3 and Supplementary Figure 3. Twenty (83%) patients had detectable ctDNA pre‐treatment, with a median of 2 (range 1–7) confirmed somatic mutations (Supplementary Table 3). Overall, 47% (*n* = 67) of the targeted somatic mutations were detected in plasma, either in the pre‐treatment sample or any of the serially collected samples (Figure 2). The number of somatic tumour mutations targeted was similar for ctDNA‐positive and ctDNA‐negative patients (median 6 vs. 5, *p* = 0.098). The most commonly mutated genes were *TP53* (*n* = 5, 21%) and *PIK3CA* (*n* = 4, 17%) (Supplementary Table 3) Patient‐ and disease characteristics were similar for ctDNA‐positive and ctDNA‐negative patients (Table 1). Notably, all patients who developed PD and/or died were ctDNA‐positive.

### 3.4 ctDNA dynamics are associated with progression‐free survival

The total ctDNA level (sum of ctDNA molecules for all mutations) decreased for all patients at C1d15, ranging from a minimum reduction of 40% to undetectable levels in 7 (35%) patients. Due to a protocol amendment, C2d1 samples were only available for 15 patients. In 7 (47%) of these patients, the total ctDNA continued to decline or remained undetectable at C2d1, while 8 (53%) showed increasing levels (Supplementary Figure 2). The median PFS was potentially shorter for patients with rising ctDNA at C2d1, but the difference was not statistically significant (7.8 vs. 25.6 months, *p* = 0.13). Patients, in whom ctDNA became undetectable at some time point (*n* = 12, 60%) exhibited longer PFS compared to those in whom ctDNA remained detectable throughout (*n* = 8, 40%) (25.6 vs. 7.8 months, *p* = 0.024).

### 3.5 ctDNA dynamics predict progressive disease

Out of the 13 patients who experienced PD, 12 (92%) showed rising ctDNA levels, either before (*n* = 7) or at (*n* = 5) PD. One patient (7.7%), in whom only one mutation was monitored, experienced PD without rising ctDNA. A borderline significant difference in median PFS was observed between those with a ctDNA rise prior to PD and those with a rise at PD (13.4 months vs. 5.5 months, *p* = 0.053). At PD, increased ctDNA levels were observed for 38 of the 40 mutations (95%) detected in the pre‐treatment samples. In addition, two patients (107 and 114) had one mutation each not detected pre‐treatment appearing at or prior to PD. The time point at which ctDNA levels rose varied between patients and among mutations for a given patient (Supplementary Table 3). The median lead time from the first ctDNA rise to PD was 114 days (range 0–450) (Figure 2). Rising ctDNA was also observed in two patients without PD during the follow‐up period. Patient 125 showed reappearing ctDNA for one of four mutations at Scan 4, but it was undetectable at Scans 5–7. In patient 206, ctDNA for one of six mutations reappeared at Scan 4, was undetectable at Scan 5, and reappeared again at Scan 6. We conducted a post hoc sensitivity analysis using both higher and lower thresholds for ctDNA rise, which had minimal impact on the results (Supplementary Tables 4A, B, and C). While the protocol stipulated follow‐up samples for ctDNA analysis to be collected during the on treatment phase of the CDK4/6i treatment cycle, 8 samples were collected off‐treatment. The rise in ctDNA observed at some of these time points was substantial and most likely due to PD rather than the CDK4/6 dosing schedule (Supplementary Table 3).

### 3.6 Circulating tumour DNA‐guided radiological follow‐up reduces imaging requirements

We investigated the potential of ctDNA monitoring to direct imaging among patients with detectable ctDNA pre‐treatment (*n* = 20). After the baseline scan, there were a total of 105 imaging time points, with corresponding ctDNA results for 104. A total of 71 scans were classified as ‘unnecessary’, whereas 33 were classified as ‘necessary’. If this approach had been used to guide imaging, 68% of the scans performed would have been avoided (Supplementary Table 4A, B, and C).

## 4 DISCUSSION

We present an experimental approach for personalised and ultrasensitive ctDNA analysis, aimed for tailoring imaging schedules in MBC. The results from this exploratory analysis show that around two‐thirds of the scans performed in ctDNA‐positive patients could potentially have been avoided using this approach.

Several studies on ctDNA monitoring in MBC have demonstrated that patients with detectable ctDNA often show increasing ctDNA levels prior to or at PD.^14^, ^15^, ^16^, ^17^, ^18^, ^19^ In the proof‐of‐concept study by Dawson et al., ctDNA was detected in 58% (*n* = 30) of the initial patient cohort, with rising ctDNA levels observed in 89% (*n* = 17) of the patients with PD.^14^ Hrebien et al. identified trackable mutations in 78% (*n* = 50) of the patients, of which 84% (*n* = 42) had detectable ctDNA. Monitoring of ctDNA every 4 weeks until PD revealed diverse patterns of ctDNA dynamics.^15^ Chin et al. analysed ctDNA in a cohort similar to ours and ctDNA was detected in 61% (*n* = 20) of the patients at some time point during the follow‐up, with increasing ctDNA levels observed in 75% (*n* = 15) of the patients with PD.^16^ Darrigues and colleagues identified trackable mutations in 41% (*n* = 25) of the patients, in whom ctDNA was detected in 84% (*n* = 21). At PD, 79% (*n* = 11) of the patients showed higher ctDNA levels compared to the 30‐day sample.^17^ Liu et al. analysed ctDNA in a heterogenous cohort of MBC patients and found rising ctDNA and/or new mutations in 78% (*n* = 45) of the patients with PD.^18^ These studies exhibit considerable variability in the method employed to analyse ctDNA, with the rationale behind the definition used for determining ctDNA rise rarely presented. Some report ctDNA as MAF, while others use molecules/mL. The sensitivity to detect ctDNA is highly dependent on which method is used to identify trackable mutations, the number of mutations monitored, and the sensitivity of the method used for ctDNA analysis. In addition, ctDNA levels may be affected by the treatment delivered,^20^ while the sampling frequency can impact the lead time from first ctDNA rise to PD. Consequently, it is difficult to directly compare studies, and for the same reason, it will be challenging to establish uniformly accepted criteria for ctDNA progression without future standardisation.

Our analytical workflow for ctDNA analysis has two major advantages: (1) a tumour‐informed approach with personalised multiplexed ctDNA panels designed from mutations identified in tissue, and (2) utilisation of an ultrasensitive sequencing method that allows us to reliably detect ctDNA with MAF around 0.1%. One strategy to further increase the sensitivity of ctDNA analysis is to monitor even more mutations in plasma using SiMSen‐Seq. In our workflow, this can be achieved by applying whole genome or exome sequencing instead of targeted gene panel sequencing when analysing tumour tissue to identify additional mutations to monitor. However, personalised ctDNA panels cannot be used to detect new mutations that emerge during disease progression. Detection of such *de novo* mutations requires plasma cell‐free DNA analysis using broad gene panels, which substantially increases costs due to the need for repeated sampling. Although the number of detected genomic alterations has been shown to increase during tumour progression,^18^, ^21^, ^22^ potentially selected by therapy pressure, the mutations present at baseline usually persist and contribute to increasing ctDNA levels at PD. This is supported by our findings where ctDNA levels for 95% of the mutations detected pre‐treatment had risen at the time of PD. It is crucial to note that any method must comply with regulatory requirements before implementation in clinical practice and joint consensus recommendations for ctDNA assay validation were recently published.^23^

We observed different patterns of ctDNA dynamics preceding PD, as previously described by others.^14^, ^15^, ^16^ ctDNA decreased to undetectable levels in more than half of the patients and most patients with PD showed substantial increase in ctDNA levels. Multiple definitions have been reported for molecular progression, each having its advantages and disadvantages.^24^ In our work, rising ctDNA was not used to define molecular progression but as an indicator to resume imaging. We prioritised sensitivity over specificity since a false positive would result in the resumption of scans rather than a change in treatment. The selected cut‐off values for ctDNA rise were robust in our setting, but larger patient cohorts are needed to fine‐tune the criteria.

Rising ctDNA coincided with or preceded PD in 92% of ctDNA‐positive patients, despite only monitoring a median of 2 mutations per patient. Notably, 95% of the mutations detected pre‐treatment exhibited elevated ctDNA levels at the time of PD, indicating that the mutations tracked were representative of the patient's metastatic disease and that the number of mutations needed to monitor is relatively low. However, additional patients and samples need to be studied to determine the minimum number of mutations required to accurately guide imaging schedules. The four patients without detectable ctDNA had similar patient and tumour characteristics as the ctDNA‐positive patients, and there was no difference in the number of somatic mutations included in the ctDNA panels. Although it is unlikely that these mutations are ‘false’, we cannot, at this stage, rule out that they may not be fully representative of their metastatic disease. The inability to detect ctDNA can also be due to low ctDNA levels, which could potentially be addressed by monitoring a higher number of mutations per patient. The absence of PD among the ctDNA‐negative patients suggests a more indolent disease, supported by previous studies showing that ctDNA‐negativity is associated with better survival outcomes.^25^ Longer follow‐up time will inform whether the ctDNA‐negative patients will exhibit detectable ctDNA at the time of PD.

In summary, this exploratory analysis shows that personalised and ultrasensitive ctDNA monitoring has the potential to substantially reduce imaging requirements among MBC patients. This concept, including criteria to define ctDNA rise, will be further investigated in the extended study cohort upon completion of the PDM‐MBC study, also including a cost‐effectiveness analysis of ctDNA‐guided imaging. Until then, we encourage others to apply our suggested approach to similar patient cohorts.

## AUTHOR CONTRIBUTIONS

**Pia Mouhanna:** Methodology; data curation; writing – original draft; writing – review and editing; visualization; project administration; investigation; validation; formal analysis. **Anders Ståhlberg:** Methodology; supervision; funding acquisition; investigation; formal analysis; resources; writing – review and editing. **Daniel Andersson:** Methodology; writing – review and editing. **Ahmed Albu‐Kareem:** Resources; writing – review and editing. **Ellinor Elinder:** Resources; writing – review and editing. **Olle Eriksson:** Formal analysis; writing – review and editing. **Amy Kavanagh:** Project administration; writing – review and editing. **Anikó Kovács:** Methodology; writing – review and editing. **Karolina F. Larsson:** Writing – review and editing; resources. **Barbro Linderholm:** Writing – review and editing; resources. **Monika Uminska:** Writing – review and editing; resources. **Tobias Österlund:** Software; writing – review and editing. **Sacha J. Howell:** Conceptualization; methodology; investigation; formal analysis; supervision; funding acquisition; writing – review and editing; resources. **Maria Ekholm:** Conceptualization; methodology; investigation; formal analysis; supervision; funding acquisition; visualization; project administration; writing – review and editing; writing – original draft; resources.

## FUNDING INFORMATION

ALF grants, Region Östergötland: RÖ‐985095, The Swedish state under the agreement between the Swedish government and the county councils, the ALF‐agreement (ALF), Region Västra Götaland, Sweden: 965065, The Christie Charity: 1201654, Foundation Assar Gabrielsson: FB20‐09 : FB21‐29 : FB22‐08, Foundation for Clinical Cancer Research in Jönköping: 180424‐2 : 191111‐1 : 20201116‐1, Futurum – the Academy for Health and Care: FUTURUM‐809561 : FUTURUM‐939920, Gunnar Nilsson Cancer Foundation: 2019‐27‐147, Kronoberg Cancer Foundation: 2019, Medical Research Council of Southeast Sweden: FORSS‐930630 : FORSS‐989664 : FORSS‐995301, Percy Falk Foundation, Sjöberg Foundation: 2022‐01‐11:4, Swedish Breast Cancer Association: F2018‐03 : F2019‐005, Swedish Cancer Society: 22‐2080 : 23 2760 Fk, Sweden’s Innovation Agency: 2020‐04141, Swedish Research Council: 2021‐01008, Swedish Society for Medical Research: P18‐0063, Swedish Society of Medicine: SLS‐934348, Swedish Society of Oncology: Scholarship 2022, and Wilhelm and Martina Lundgren Research Foundation: 2023‐GU‐4185. The funders played no role in study design, data collection, analysis and interpretation of data, or the writing of this manuscript.

## CONFLICT OF INTEREST STATEMENT

Anders Ståhlberg is a shareholder of SiMSen Diagnostics and iScaff Pharma, a shareholder and board member of Tulebovaasta, and co‐inventor of the SiMSen‐Seq technology, patent (U.S. #15/552,618). Anikó Kovács has participated in Advisory Boards for Pfizer. Karolina F Larsson has participated in Advisory Boards for Pfizer. Barbro Linderholm has participated in Advisory Boards for Pfizer and Eli Lilly. Sacha J. Howell has participated in Advisory Boards for Eli Lilly, has received Speaker Fees from Pfizer, Eli Lilly, and Novartis, and has a Shared Working Agreement with Eli Lilly. Maria Ekholm has participated in Advisory Boards for Pfizer, Novartis, and Eli Lilly. The other authors declare no financial or non‐financial competing interests relevant to this work.

## ETHICS STATEMENT

The study was approved by the ethical review authorities in the United Kingdom (19/WS/0012) and Sweden (2019‐05238). All patients provided written informed consent to participate, and the study was carried out in accordance with the Declaration of Helsinki.

## Supporting information


Data S1

Table S5

## Data Availability

The datasets used or generated in this study are available from the corresponding author upon reasonable request.
