# Supplementary material for: Integration of personalised ultrasensitive ctDNA monitoring of patients with metastatic breast cancer to reduce imaging requirements
Source: Int J Cancer. 2024 Dec 18;156(8):1509–17. doi: 10.1002/ijc.35292 (PMC11826139; doi:10.1002/ijc.35292)
Supplement: Supplementary file 1 — Data S1 [file IJC-156-1509-s002.pdf]

SUPPLEMENTARY MATERIAL

TITLE

Integration of personalised ultrasensitive ctDNA monitoring of patients with metastatic breast cancer to reduce imaging requirements

AUTHORS

Pia Mouhanna, Anders Ståhlberg, Daniel Andersson, Ahmed Albu-Kareem, Ellinor Elinder, Olle Eriksson, Amy Kavanagh, Anikó Kovács, Karolina F Larsson, Barbro Linderholm, Monika Uminska, Tobias Österlund, Sacha J Howell, and Maria Ekholm

TABLE OF CONTENT

Supplementary Methods
- 2.1 Patient cohort and study design 2
- 2.2 Blood sampling and cell-free DNA extraction 2
- 2.3 Targeted tumour tissue sequencing 2
- 2.4 Design of personalised ctDNA panels 3
- 2.5 Personalised ctDNA analysis 3
- 2.6 Sequencing and data analysis 4
- 2.7 Circulating tumour DNA data analysis 4
- 2.8 Statistical analysis 5
- Eligibility criteria 6
- Instructions for tissue request 7
- Definition of the limit function used to define ctDNA rise 8

Supplementary Tables
- Supplementary Table 1. Patient and tumor characteristics 9
- Supplementary Table 2. Technical evaluation of ctDNA panels 10
- Supplementary Table 3. Summary of experimental data for ctDNA analysis for detected mutations presented along with imaging results 12
- Supplementary Table 4A. Original and altered criteria and limit function used to define ctDNA rise 22
- Supplementary Table 4B. Time point for ctDNA rise using original and altered criteria .. 22
- Supplementary Table 4C. Outcomes using original and altered criteria for ctDNA rise 22
- Supplementary Table 5. Sequencing, coverage and quality stastistics. This table is provided separately as an excel file.

Supplementary Figures
- Supplementary Figure 1. Evaluation of ctDNA panels based on fragment analysis 23
- Supplementary Figure 2. Early ctDNA dynamics 24
- Supplementary Figure 3. Circulating tumour DNA levels and imaging results for each of the patients with detectable ctDNA 25

References
- Supplementary reference list 29

Supplementary Methods

2.1 Patient cohort and study design

The PDM-MBC study prospectively recruited 97 patients diagnosed with advanced estrogen receptor (ER) positive and human epidermal growth factor receptor 2 (HER2) negative breast cancer, eligible for 1st line endocrine therapy including an aromatase inhibitor (+/- a gonadotropin-releasing hormone analogue if pre/perimenopausal) and a cyclin dependent kinase 4/6 inhibitor (CDK4/6i) (ribociclib or palbociclib) across one site in England and five sites in Sweden. Patients were recruited between. June 2019 and March 2023 and follow-up will continue until progressive disease (PD) or May 2026, whichever comes first. The CDK4/6i dosing schedule included daily administration for three weeks (day 1 to 21) followed by one week off treatment (day 22 to 28) and dose adjustments were made as per standard of care. Change to abemaciclib was allowed if ribociclib or palbociclib was not tolerated due to toxicity. Termination of CDK4/6i was allowed for patients unable to tolerate any CDK4/6i. Computed tomography (CT) of thorax, abdomen and pelvis, assessed according to response evaluation criteria in solid tumours (RECIST) 1.1 [1] was performed every three to four months, or earlier if PD was clinically suspected. Magnetic resonance imaging (MRI) of representative metastases was mandatory for those with bone only or bone-predominant disease lacking target lesions by RECIST 1.1. Blood samples were collected pre-treatment, after 2 weeks (C1d15), after 4 weeks just before the initiation of cycle 2 (C2d1), and thereafter concurrently with imaging until PD. The protocol stipulated imaging and follow-up samples to be carried out on while on CDK4/6i (day 15 +/- 6 days). In case samples were missed or could not be collected for other reasons, study participants were asked to attend for study samples as soon as possible. Analysis of ctDNA was not conducted in real-time, and the results were not communicated to the treating clinician.

2.2 Blood sampling and cell-free DNA extraction

Blood samples were collected into 10 mL BD Vacutainer K2 EDTA tubes (Becton Dickinson). and centrifuged at room temperature for 10 min at 2,000 g within 1 hour of collection. The plasma was transferred to a BD Falcon tube (Becton Dickinson) and centrifuged a second time at room temperature for 10 min at 2,000 g and transferred to 2.0 mL aliquots. Plasma and separated buffy coat were stored at a maximum of -70 °C. Cell-free DNA was extracted from 4 mL plasma using either the QIAsymphony DSP Circulating DNA Kit, or manually using the QIAmp Circulating Nucleic Acid Kit (both Qiagen). Extracted DNA was stored at -80 °C. The cell-free DNA concentration was quantified with a Qubit fluorometer (Thermo Fischer Scientific) and samples < 1 ng cell-free DNA/µL were concentrated using the Vivacon 500 concentrator (Sartorius) at 5,000 g with an elution volume of 10 µL. DNA from buffy coat was extracted with the QIAmp DNA Blood Mini Kit (Qiagen), according to the manufacturer's instructions and stored at -20 °C.

2.3 Targeted tumour tissue sequencing

Archived tumour tissue, either from a metastasis or the primary tumour, was requested as sections mounted on glass slides, or as tumour blocks from which sections were cut at the central laboratory. Details on the instructions for tissue request is provided at page 7. DNA was extracted using the GeneRead DNA FFPE Kit (Qiagen), according to the manufacturer's instructions. The DNA concentration was quantified with a Qubit fluorometer and stored at -20 °C. Extracted DNA was sequenced at Genomic Medicine Sweden, Gothenburg, Sweden, using a gene panel for solid tumours covering 544 genes and ~1,600,000 bases of coding sequence [2].

2.4 Design of personalised ctDNA panels

Data processing and variant calling of the panel sequencing data was performed using the Genomic Medicine Sweden inhouse bioinformatics pipeline. Variant annotation was performed using Qiagen Clinical Insight, QCI-T translational (Qiagen). All identified mutations were manually inspected in the Integrative Genomics Viewer version 2.13.1 with the provided bam files in human genome 19. Single nucleotide variants and short insertions and deletions with tumour mutant allele frequency (MAF) ranging from 5%–75% were selected. Mutations located at the start or end of reads, positions with < 5 total reads, and insertions or deletions covering more than 15 nucleotides were omitted. Known single nucleotide polymorphisms and genes identified as bioinformatically problematic, such as *HLA-A* and *HLA-B,* were excluded. Remaining mutations were used to design personalised ctDNA panels using PanelPlex (DNA Software). A schematic overview of the experimental workflow for personalised ctDNA analysis is shown in Manuscript Figure 1A.

2.5 Personalised ctDNA analysis

We applied SiMSen-Seq to quantify ctDNA levels in plasma [3] and the SiMSen-Seq protocol is outlined in Manuscript Figure 1B. Briefly, barcoding PCR was performed in 15 µL reactions, containing Platinum SuperFi II Buffer (1X, Thermo Fischer Scientific), Platinum SuperFi II DNA polymerase (0.01 U, Thermo Fisher Scientific), deoxyribonucleotide triphosphate (0.2 mM, Sigma-Aldrich), target primers (40 nM, either oPools or Ultramers, both Integrated DNA Technologies), L-carnitine inner salt (0.5 M, Sigma-Aldrich) and target DNA. For ctDNA panel evaluation we used 20 ng DNA from patient-specific buffy coat or commercial reference DNA (human genomic DNA, Roche Diagnostics). For plasma samples we used ≤ 40 ng ~~cfDNA~~ cell-free DNA per analysis. The following thermal program was used on a T100 Thermal cycler (Bio-Rad Laboratories, Inc): 3 min at 98 °C, 3 cycles of amplification (98 °C for 10 s, 60 °C for 6 min, 72 °C for 30 s), 15 min at 65 °C and 15 min at 95 °C. At the start of the 15 min incubation step at 65 °C, 30 µL TE-buffer (pH 8.0, Thermo Fisher Scientific) supplemented with *Streptomyces griseus* protease (45 ng/µL, Sigma-Aldrich) was added to terminate the reaction. One third of the reaction volume was transferred to the adapter PCR performed in 60 µL reactions, containing Platinum SuperFi II PCR Master Mix (1x, Thermo Fischer Scientific) and Illumina adapter primers (400 nM, desalted, Integrated DNA Technologies) [18]. The following thermal program was used for the adapter PCR on a T100 Thermal cycler: 98 °C for 3 min, 27 cycles of amplification (98 °C for 10 s, 80 °C for 1 s, 72 °C for 30 s, 76 °C for 30 s, with a ramping rate of 0.2 °C/s between 80 °C, 72 °C and 76 °C).

Library quality and concentration were evaluated on a 5200 Fragment Analyzer System using the HS NGS Fragment kit (1 – 6000 bp, both Agilent Technologies), according to the manufacturer's instructions. To assess the performance of the personalised ctDNA panels, we constructed SiMSen-Seq libraries using patient-specific DNA from buffy coat and commercial reference DNA. If no buffy coat was available, only commercial reference DNA was used. The yield was assessed with parallel capillary electrophoresis using a pre-defined specificity criteria of ≥10 nmol/L specific library product using ProSize Data Analysis Software version 3.0.1.6. To identify germline mutations, ctDNA panels were sequenced with patient-specific buffy coat DNA. For patients with no buffy coat available, mutations detected in plasma with a consistent MAF around 50 % and 100% at all time points were considered as germline [4] and excluded from subsequent data analysis.

Individual libraries were pooled based on input DNA and panel size in the barcoding PCR to provide similar sequencing depth. Pooled libraries were purified using a Pippin Prep instrument with the 2% agarose gel cassette (both Sage Science), according to the manufacturer's instructions with a target range between 210 and 300 bp.

2.6 Sequencing and data analysis

The final sequencing pool was quantified on a CFX384 Touch Real-Time PCR Detection System (Bio-rad Laboratories, Inc) in a 10 µL reaction, containing TATAA SYBR GrandMaster Mix Low Rox (1x, TATAA Biocenter), Illumina quantitative PCR primers for library quantification (400 nM of each primer, 5'-AATGATACGGCGACCACCGA-3' and 5'-CAAGCAGAAGACGGCATACGA-3') and 2 µL purified library pool. The concentration was determined using a standard curve with adjustments to the average library size. The final pool concentrations were adjusted to 1.2-1.8 pM and supplemented with PhiX Control v3 (20%, Illumina). Sequencing was performed on either a MiniSeq using a Mid or High Output Reagent Kit or on a NextSeq using a Mid output Reagent Kit (Illumina), all with single-end and 150 bp sequencing.

Raw sequencing reads were bioinformatically processed using UMIErrorCorrect version 0.24 [5]. Briefly, sequencing reads were collapsed into consensus reads, requiring ≥ 3 reads for each unique molecular identifier family. A single nucleotide mutation was considered true if detected in ≥ 2 consensus reads and with ≥ 0.1% MAF, while insertions and deletions only required one consensus read. In SiMSen-Seq, about two consensus reads correspond to one DNA molecule. As a technical quality control after sequencing, the number of detected consensus reads was compared with input DNA. The background noise at all nucleotide positions with mutations was also controlled using data from either patient-specific DNA from buffy coat or commercial reference DNA.

Sequencing detected a median of 1433 molecules (range 402 to 3502) per ctDNA panel in control samples (buffy coat and commercial reference DNA). Of the assays targeting 227 mutations in total, 11 (4.8%) failed to generate any sequencing data. One hundred and forty-four (63%) of the targeted mutations were considered somatic (median 5.5 per ctDNA panel, range 1 to 13) and 72 (32%) germline (median 2.5 per ctDNA panel, range 0 to 7).

2.7 Circulating tumour DNA data analysis

Circulating tumour DNA was reported both as mutant molecules per mL plasma and as the fraction of mutant to wild-type copies, i.e., MAF. Circulating tumour DNA molecules/mL plasma is not affected by the amount of wild-type cell-free DNA, whereas MAF has the benefit of being less affected by preanalytical steps and sequencing depth [6, 7]. To guide imaging needs, we prioritised high sensitivity, while accepting lower specificity. To determine whether the ctDNA level for each mutation had increased at a specific time point, the level (molecules/mL) was compared to its lowest previously observed level ($ctDNA_{nadir}$), disregarding the C1d15 time point. The reason for excluding the C1d15 time point was due to the ctDNA rise frequently observed from C1d15 to C2d1, as a consequence of the intermittent CDK4/6i dosing schedule. The level of ctDNA was considered to rise if any of the following criteria were fulfilled: (1) reappearance of ctDNA if $ctDNA_{nadir}$ was below the threshold for detection, or detection of ctDNA if previously undetected; (2) > 3-fold increase if $ctDNA_{nadir} \leq 1$ molecule/mL plasma; (3) > 2-fold increase if $ctDNA_{nadir} \geq 1$–5 molecules/mL; (4) > 1.5-fold increase if $ctDNA_{nadir} >$ 5 molecules/mL. To facilitate a smooth transition between the criteria for the various nadir levels, we defined a limit function, expressed as $ctDNA_{nadir} \times (1.5 + 1.47 \times e^{-0.39 \times ctDNAnadir})$. A description of the limit function is provided at page 8. Rising ctDNA was considered to exist if the ctDNA level for ≥1 mutation exceeded the value of the limit function. The lead time from ctDNA rise to PD was calculated as the time from detection of first ctDNA rise to PD. The underlying reason for the non-linear definition of ctDNA rise is to account for sampling ambiguity when handling a small number of molecules. A post hoc sensitivity analysis was conducted, applying both higher and lower thresholds for ctDNA rise (Supplementary Tables 4A-C).

2.8 Statistical analysis

Summary statistics were provided for patient and tumour characteristics. Fisher's exact test was used to compare categorical variables, while median test and Mann Whitney U-test was used to compare continuous variables between cDNA-positive and ctDNA-negative patients. Distant recurrence-free interval was defined as the time from diagnosis of the primary cancer to the diagnosis of advanced disease. Progression-free survival (PFS) was defined as the time from study consent to PD or death from any cause, using October 1, 2023 as data cut-off. Patients without PD were censored at the time of their last scan. Survival analysis was performed using the Kaplan–Meier method and log-rank test was employed to compare subgroups. All presented p-values are two-sided and p-values < 0.05 were considered statistically significant. Statistical analyses were performed using SPSS software version 29.0 and R software version 4.2.2.

Eligibility criteria

Inclusion criteria

- Confirmed metastatic breast cancer (stage 4) or locally advanced disease not amendable to curative resection. Patients who have locoregional disease only an in whom a response to therapy would lead to potentially curative resection are not eligible.
- ER+/HER2- breast cancer (assessed locally).
  NOTE: If immunohistochemical analysis is not available from metastatic tissue, the ER and HER2 status from the primary tumour should be used.
- For patients who have had a prior non-breast malignancy within the last 5 years (excluding in situ carcinoma of the cervix and basal cell carcinoma of the skin) biopsy of a metastatic site is required to confirm the diagnosis of metastatic ER+/HER2- breast cancer.
- Eligible for 1st line endocrine treatment with AI + CDK4/6-inhibitor according to local guidelines.
  NOTE: ≥ 12 month since termination of adjuvant AI if used. Patients may have received one prior line of chemotherapy for metastatic breast cancer but should have disease progression at study entry.
- Eastern Cooperative Oncology Group Performance Status (ECOG PS) 0-2
- Life expectancy >3 months. Age ≥ 18 years
- Metastatic disease must be radiologically assessable, by means of at least one of the following techniques: computerised tomography (CT) and/or magnetic resonance imaging (MRI), i.e. lesions can be measurable or non-measurable according to RECIST 1.1, but must be clearly evaluable according to the radiologist and/or treating physician. (Patients with bone predominant disease who lack evaluable disease on CT according to RECIST 1.1. must have serial MRI including the representative area in addition to CT thorax-abdomen-thorax).
  NOTE: Previously irradiated lesions are deemed measurable only if progression is documented at the site after completion of radiation.
- Willing and able to provide informed consent to undergo all trial procedures.

Exclusion criteria

- Known CNS metastases, carcinomatous meningitis or leptomeningeal disease unless treated with radiotherapy and symptomatically stable at least 2 weeks after discontinuation of steroids. For such patients, ongoing monitoring of CNS disease is required as standard of care. No screening is needed for asymptomatic patients.
- Concurrent disease(s) or familial, sociological or geographical condition that would, in the investigator's opinion, preclude compliance with study procedures.
- Any serious medical disorder that would compromise the patient's safety.
- Dementia, altered mental status, or any psychiatric condition that would prevent the understanding or rendering of Informed Consent.

Instructions for tissue request

Preferred tumour tissue

The order of preference for tumour tissue samples, with 1 being the most preferred, is:

1. Biopsy or surgical specimen from a non-bone metastasis
2. Surgical specimen from the primary tumour unless neoadjuvant therapy undertaken.
3. Core biopsy from the primary tumour
4. Surgical specimen from primary tumour after neoadjuvant therapy
5. Biopsy or surgical sample from a bone metastasis

Selection of representative tissue

The quality of molecular test results is highly dependent upon proper specimen collection and handling procedures. It is therefore of utmost importance that the tissue contains a representative area of invasive tumour cells.

- The fraction of invasive neoplastic cells should preferably be ≥20%, but must exceed ≥10% in part of (see below) or the whole section
- The size of the region where the neoplastic cells are located should be at least 5mm2 (not applicable for core biopsies)

Note: Areas with necrosis should be avoided

Note: The fraction of invasive neoplastic cells is defined as the ratio of invasive neoplastic cell nuclei to the total number of cell nuclei (invasive neoplastic cells and non-invasive neoplastic cells and non-neoplastic cells) in part of (see below) or the whole section

Provision of formalin-fixed paraffin-embedded (FFPE) tissue material

- If the tumour tissue is ≥ 25 mm2 **and** includes ≥20% neoplastic cells => **ten** sections (Superfrost Plus (+) slides or similary positively charged slides only) of 5µm each should be sent, of which the last one should be stained with hematoxylin and eosin.
- If the tumour tissue is < 25 mm2 **or** includes 10-20% neoplastic cells => **twenty** sections (Superfrost Plus (+) slides or similary positively charged slides only) of 5µm each should be sent, of which the last one should be stained with hematoxylin and eosin.

General cutting instructions

FFPE sections (put on Superfrost Plus (+) or similarly positively charged slides and unbaked at 5 microns thick) should be obtained in a manner preventing cross-contamination, since the material is going to be used for subsequent molecular analyses.

Appropriate measures should be taken to prevent cross-contamination between material from different patients:

- Use for each FFPE-block a clean new blade.
- Clean the microtome with ethanol 100% (especially the specimen holder) from all debris when changing FFPE-blocks between patients.
- Use fresh and clean warm and fresh cold-water baths to stretch your cut unstained sections to prevent cross contamination by floaters and debris from other paraffin blocks.
- Wear gloves and change them between samples.

Definition of the limit function used to define ctDNA rise

The three criteria used for detecting a rise in ctDNA at various nadir levels are fixed at the ends of the line segment (segments 2 to 4 in Manuscript Figure 1C). To achieve a continuous smooth transition between these criteria, we defined a function to replace the criteria. Replacing $ctDNA_{nadir}$ by x for short, the following targets had to be fulfilled:

1. it should start at origo, or in other words, f(0)=0
2. it should pass the average of the two points in the discontinuity at x=1, or f(1)=2.5
3. it should pass the average of the two points in the discontinuity at x=5, or f(5)=8.75
4. it should asymptotically narrow in on 1.5 times $ctDNA_{nadir}$ at higher levels, or f(x)=1.5·x for large x, for example f(15)=22.5

    A structure for a function that meets these 4 targets can be composed as f(x)=x·(1.5 + *ADJ*) where *ADJ* serves as a low x-adjustment that is allowed to be high at x=0 but needs to level off up to roughly x=10. An *ADJ* with these properties can be written as a·exp(b·x) where a is the height of the adjustment at x=0 and b defines how rapidly the adjustment levels of at higher x. The parameters are not mathematically restricted, but they can be interpreted as above only if a>0 and b<0.

    f(0)=0 holds for any a and b and will not be mentioned when discussing the choice of a and b below. The parameters can be searched for or found by non-linear regression. With a=1.414 and b=-0.346, it is observed that f(1)=2.50, f(5)=8.75 and f(15)=22.62 (rounded to 2 decimals) which are all close to their respective targets.

5. the deviation between the function and the criteria, including its sign, should be close to zero on average. With a=1.414 and b=-0.346 the function takes generally a little too large values over the range 0—15. The parameters were adjusted to 1.46 and -0.39. By that the average deviation between the function and the criteria was roughly halved and the function almost meet the targets 1 to 3 with f(1)=2.49, f(5)=8.54 and f(15)=22.56 (rounded to 2 decimals). Adjusting the parameters to a=1.46 and b=-0.39 has pushed the function closer to the criteria on average but causes the function to miss the target 3 slightly.

Supplementary Tables

Supplementary Table 1. Patient and tumor characteristics

Patient	101	104	107	109	110	113	114	115	116	118	119	121	123	124	125	126	201	202	203	205	206	207	208	213
Age, years	62	45	46	62	47	49	55	56	33	56	28	55	34	54	44	73	50	41	81	65	84	72	66	57
Performance status [a]	1	0	0	0	0	0	0	0	0	0	1	1	1	0	0	1	0	1	2	1	1	0	0	0
Recurrent/*de novo* disease	*dn*	*dn*	R	R	*dn*	*dn*	R	*dn*	*dn*	R	*dn*	R	R	*dn*	R	R	*dn*	R	R	R	*dn*	R	R	R
Distant recurrence-free interval, years	NA	NA	5.8	13.2	NA	NA	12.1	NA	NA	5.2	NA	22.7	0.3	NA	11.2	22.8	NA	5.0	13.0	20.1	NA	15.9	10.5	7.7
Previous neoadjuvant/ adjuvant systemic therapy																								
Chemotherapy			No	Yes			Yes			Yes		Yes	No		Yes	Yes		Yes	No	Yes		Yes	No	No
Endocrine	NA	NA	Yes	Yes	NA	NA	Yes	NA	NA	Yes	NA	Yes	Yes	NA	No	Yes	NA	Yes	Yes	Yes	NA	Yes	No	Yes
Bisphosphonates			No	No			No			No		No	No		No	No		No	No	No		No	No	No
Disease stage according to TNM [b]	III	IV	IV	IV	IV	IV	IV	IV	IV	IV	IV	IV	IV	IV	IV	IV	IV	IV	IV	IV	IV	IV	IV	IV
Sites of metastatic lesions																								
Breast/chest wall	X	X			X	X	X		X		X			X			X							
Lymph nodes	X	X		X	X		X		X			X	X	X		X	X							
Bone		X	X		X	X		X	X	X	X	X	X	X	X		X	X	X		X		X	
Liver			X							X	X							X		X		X		
Lung				X							X					X	X	X			X		X	
Other non-visceral [d]												X												
Other visceral [e]																		X		X				X
Measurable disease [c]	Yes	Yes	Yes	Yes	Yes	No	Yes	No	Yes	Yes	Yes	Yes	Yes	Yes	No	No	Yes	Yes	No	Yes	No	Yes	Yes	No

Abbreviation: dn, de novo; ECOG, Eastern Cooperative Oncology Group; R, Recurrent; NA, not applicable; RECIST, Response Evaluation Criteria In Solid Tumours.

[a] According to ECOG [8]

[b] TMN Classification for malignant tumors [9]

[c] According to RECIST 1.1 [1]

[d] Includes metastases in skin or soft tissue

[e] Includes metastases in bladder, spleen, pancreas, uterus/ovaries, or peritoneum

Supplementary Table 2. Technical evaluation of ctDNA panels

**Patient**	**101**	**104**	**107**	**109**	**110**	**113**	**114**	**115**	**116**	**118**	**119**	**121**
Recurrent/*de novo* disease	*dn*	*dn*	R	R	*dn*	*dn*	R	*dn*	*dn*	R	*dn*	R
Tumour tissue used for mutation identification[a]	Primary tumour	Primary tumour	Primary tumour	Metastasis	Primary tumour	Primary tumour	Primary tumour	Primary tumour	Primary tumour	Metastasis	Metastasis	Metastasis
Detected mutations, n	27	24	35	28	20	18	28	80	14	18	13	19
Mutations included in the ctDNA panel, n	15	11	8	21	10	6	11	7	5	11	7	10
DNA source for ctDNA panel evaluation	crDNA [a]	crDNA [a]	Buffy coat	Buffy coat	crDNA [a]	Buffy coat	Buffy coat	Buffy coat	Buffy coat	Buffy coat	Buffy coat	Buffy coat
Detected molecules, n, median (min to max)	NA	NA	1901 (707 to 2514)	1943 (0 to 2878)	NA	1827 (79 to 2543)	1218 (0 to 2649)	2192 (846 to 3639)	1046 (0 to 1417)	978 (220 to 2421)	1433 (1094 to 2745)	3169 (677 to 4592)
Failed assays, n	NA	NA	0	2	NA	0	1	0	1	0	0	0
Germline mutations, n	2 [b]	1 [b]	2	7	6 [b]	4	2	1	3	6	3	6
Somatic mutations, n	13	10	6	12	4	2	8	6	1	5	4	4
**Patient**	**123**	**124**	**125**	**126**	**201**	**202**	**203**	**205**	**206**	**207**	**208**	**213**
Recurrent/de novo disease	R	*dn*	R	R	*dn*	R	R	R	*dn*	R	R	R
Tumour tissue used for mutation identification	Metastasis	Primary tumour	Primary tumour	Primary tumour	Primary tumour	Metastasis	Metastasis	Metastasis	Primary tumour	Metastasis	Primary tumour	Metastasis
Detected mutations, n	13	15	15	28	20	13	18	54	16	19	27	18
Mutations included in the ctDNA panel, n	9	10	8	13	5	4	9	10	9	8	12	8
DNA source for ctDNA panel evaluation	Buffy coat	Buffy coat	Buffy coat	Buffy coat	Buffy coat	Buffy coat	Buffy coat	Buffy coat	Buffy coat	Buffy coat	Buffy coat	Buffy coat
Detected molecules, n, median (min to max)	3502 (1902 to 5277)	1697 (684 to 2845)	2310 (1560 to 3433)	1139 (0 to 4154)	590 (237 to 779)	402 (0 to 822)	588 (0 to 816)	755 (0 to 1407)	611 (205 to 893)	977 (146 to 1151)	2241 (1069 to 3799)	2526 (1876 to 3484)
Failed assays, n	0	0	0	1	0	1	3	1	0	1	0	0
Germline mutations, n	4	5	3	4	0	2	1	2	2	1	4	3
Somatic mutations, n	5	5	5	8	5	1	5	7	7	6	8	5

Abbreviations: ctDNA, circulating tumour DNA; crDNA, commercial reference DNA; dn, de novo; R, recurrent; *; NA, not applicable.*
[a] the ctDNA-panel was only evaluated on the Fragment Analyzer System
[b] germline mutations were identified from plasma cell-free DNA analysis

Supplementary Table 3. Summary of experimental data for ctDNA analysis for detected mutations presented along with imaging results

Patient 101		Pre-T	C1d15	C2d1	Scan 1	Scan 2	Scan 3		
	Days from C1d1	0	15		77	161	252		
	Plasma, mL	1.60	2.05		2.55	1.90			
	Cell-free DNA, ng	19.4	14.4	Not in protocol	18.8	14.4	Missing		
	Amount of DNA analysed, ng	15.5	11.5		15.0	11.5			
Mutation 1 *FAT3*	Total number of molecules detected[a]	187	42		167	164			
	ctDNA, molecules/mL plasma	1.56	0		0	0			
	MAF, %	1.07	0		0	0			
Mutation 2 *RPTOR*	Total number of molecules detected[a]	942	469		840	783			
	ctDNA, molecules/mL plasma	12.9	0		0	0			
	MAF, %	1.75	0		0	0			
Mutation 3 *PTCH1*	Total number of molecules detected[a]	686	347		624	597			
	ctDNA, molecules/mL plasma	12.9	0		0	0			
	MAF, %	2.41	0		0	0			
Mutation 4 *RNF111*	Total number of molecules detected[a]	827	368		786	684			
	ctDNA, molecules/mL plasma	14.8	0		0	0			
	MAF, %	2.30	0		0	0			
Mutation 5 *TERT*	Total number of molecules detected[a]	39	14		34	28			
	ctDNA, molecules/mL plasma	1.17	0		0	0			
	MAF, %	3.85	0		0	0			
Mutation 6 *AXIN1*	Total number of molecules detected[a]	643	324		546	514			
	ctDNA, molecules/mL plasma	11.7	0		0	0			
	MAF, %	2.33	0		0	0			
	Total ctDNA, molecules/mL plasma	55.03	0		0	0			
	Imaging result				PR	PR	PR		
**Patient 104**		**Pre-T**	**C1d15**	**C2d1**	**Scan 1**	**Scan 2**	**Scan 3**	**Scan 4**	**Scan 5**
	Days from C1d1	-1	15		70	154	238	322	406
	Plasma, mL	1.70	2.05		3.30	1.75	3.40	3.10	3.75
	Cell-free DNA, ng	11.7	18.9	Not in protocol	14.8	9.13	18.8	11.6	31.5
	Amount of DNA analysed, ng	9.37	15.1		11.8	7.30	15.1	9.24	25.2
Mutation 1 *MACF1*	Total number of molecules detected[a]	144	271		234	164	325	195	534
	ctDNA, molecules/mL plasma	3.31	0.61		0	0	0	1.01	19.8
	MAF, %	3.14	0.37		0	0	0	1.29	11.1

Mutation 2 *ROS1*	Total number of molecules detected[a]	424	771		628	785	901	479	1524				
	ctDNA, molecules/mL plasma	13.6	6.71		0	0	0	2.02	69.3				
	MAF, %	4.36	1.43		0	0	0	1.04	13.6				
Mutation 3 *CTCF*	Total number of molecules detected[a]	307	601		446	432	701	388	1090				
	ctDNA, molecules/mL plasma	6.62	2.74		0	0	1.10	1.01	47.8				
	MAF, %	2.94	0.75		0	0	0.43	0.64	13.2				
Mutation 4 *WWC1*	Total number of molecules detected[a]	79	125		188	75	198	175	343				
	ctDNA, molecules/mL plasma	1.84	1.83		0	0	0	0.40	13.8				
	MAF, %	3.18	2.40		0	0	0	0.57	12.1				
Mutation 5 *PTPRC*	Total number of molecules detected[a]	363	655		447	584	735	389	1482				
	ctDNA, molecules/mL plasma	15.8	4.57		0	0	0.73	2.22	58.7				
	MAF, %	5.93	1.15		0	0	0.27	1.42	11.9				
Mutation 6 *CHD3*	Total number of molecules detected[a]	36	61		150	21	86	130	125				
	ctDNA, molecules/mL plasma	1.47	0.61		0	0	0	0.60	6.83				
	MAF, %	5.56	1.64		0	0	0	1.16	16.5				
Mutation 7 THRAP3	Total number of molecules detected[a]	323	610		457	577	735	400	1176				
	ctDNA, molecules/mL plasma	13.2	6.10		0	0	0.55	2.02	78.8				
	MAF, %	5.60	1.64		0	0	0.20	1.25	20.1				
	Total ctDNA, molecules/mL plasma	42.2	23.2		0	0	2.38	9.28	295				
	Imaging result				PR	PR	PR	PR	PD				
**Patient 107**		**Pre-T**	**C1d15**	**C2d1**	**Scan 1**	**Scan 2**	**Scan 3**	**Scan 4**	**Scan 5**	**Scan 6**	**Scan 7**	**Scan 8**	**Scan 9**
	Days from C1d1	0	15		70	176	259	343	427	553	679	799	910
	Plasma, mL	4.00	1.80		3.40	3.85		3.20	3.00	2.75	3.10	1.6	2.35
	Cell-free DNA, ng	101	28.5	Not in protocol	18.6	22.4	Missing	17.7	12.1	9.4	11.3	9.46	8.75
	Amount of DNA analysed, ng	14.7	2.60		1.70	2.04		6.44	1.10	0.94	1.03	7.57	7.00
Mutation 1 *GATA3*	Total number of molecules detected[a]	747	219		139	158		224	88	134	147	955	1147
	ctDNA, molecules/mL plasma	4.30	0		0	0		0	0	0	0	1.17	6.65
	MAF, %	0.33	0		0	0		0	0	0	0	0.16	1.09
Mutation 2 *KMT2C*	Total number of molecules detected[a]	730	131		83	201		198	81	131	92	478	497
	ctDNA, molecules/mL plasma	0	0		0	0		0	0	0	3.23	1.17	3.99
	MAF, %	0	0		0	0		0	0	0	0.11	0.31	1.51
	Total ctDNA, molecules/mL plasma	4.19	0		0	0		0	0	0	3.23	2.34	10.6
	Imaging result				SD	PR	PR	PR	PR	PR	PR	PR	PD

**Patient 109**		**Pre-T**	**C1d15**	**C2d1**	**Scan 1**	**Scan 2**	**Scan 3**	**Scan 4**	**Scan 5**	**Scan 6**	**Scan 7**	**Scan 8**	**Scan 9**	**Scan 10**	**Scan 11**	**Scan 12**	**Scan 13**
	Days from C1d1	0	15		70	154	238	338	450	567	674	799	900	1026	1138	1257	1369
	Plasma, mL	4.00	3.90		3.00		3.50	3.15	2.55	2.50	3.30	2.33	2.45	2.80	3.05	3.50	3.03
	Cell-free DNA, ng	19.1	15.9	Not in protocol	13.2	Missing	15.2	14.0	10.1	9.35	13.1	8.20	11.2	18.7	16.1	22.1	33.6
	Amount of DNA analysed, ng	15.3	12.7		10.6		12.1	11.2	8.10	7.48	10.5	6.56	8.98	15.0	12.8	17.6	26.9
Mutation 1 *FOXA1*	Total number of molecules detected[a]	401	429		297		252	162	46	51	108	343	679	1203	1089	1430	1941
	ctDNA, molecules/mL plasma	1.25	0.32		0		0	0	0	0	0	0	0	0	0	0	0
	MAF, %	1.00	0.23		0		0	0	0	0	0	0	0	0	0	0	0
	Imaging result				PR	PR	PR	PR	PR	PR	PR	PR	PR	PR	PR	PR	PR
**Patient 110**		**Pre-T**	**C1d15**	**C2d1**	**Scan 1**	**Scan 2**	**Scan 3**	**Scan 4**									
	Days from C1d1	0	16		71	154	238	322									
	Plasma, mL	3.10	3.55		3.00	3.55	3.15	1.90									
	Cell-free DNA, ng	15.7	16.0	Not in protocol	15.2	39.9	27.6	100									
	Amount of DNA analysed, ng	12.5	12.8		12.2	31.9	22.0	14.5									
Mutation 1 *TP53*	Total number of molecules detected[a]	1027	1173		870	2829	1752	744									
	ctDNA, molecules/mL plasma	5.44	1.23		0.63	0.70	1.59	1198									
	MAF, %	1.31	0.30		0.17	0.07	0.23	44.5									
Mutation 2 *NOTCH4*	Total number of molecules detected[a]	168	220		245	498	307	138									
	ctDNA, molecules/mL plasma	0.40	0.35		0.63	0	0	127									
	MAF, %	0.60	0.45		0.61	0	0	25.4									
	Total ctDNA, molecules/mL plasma	5.84	1.58		1.26	0.70	1.58	1325									
	Imaging result				SD	SD	SD	PD									
**Patient 113**		**Pre-T**	**C1d15**	**C2d1**	**Scan 1**	**Scan 2**	**Scan 3**	**Scan 4**	**Scan 5**	**Scan 6**	**Scan 7**	**Scan 8**	**Scan 9**	**Scan 10**	**Scan 11**		
	Days from C1d1	0	15	29	70	154	238	322	406	490	574	686	800	912	1024		
	Plasma, mL	3.25	2.80	2.40	2.35	3.40	3.20	2.90	3.70	3.55	2.55	2.53		2.70	3.00		
	Cell-free DNA, ng	37.6	13.1	11.9	17.1	24.5	19.3	46.2	38.3	56.1	33.6	36.7	Missing	24.2	26.7		
	Amount of DNA analysed, ng	30.0	10.5	9.50	13.6	19.6	15.4	37.0	30.6	44.9	26.8	29.4		19.4	21.4		
Mutation 1 *RUNX1*	Total number of molecules detected[a]	3059	872	884	1492	1837	1091	2906	2733	5112	861	3405		1767	1167		
	ctDNA,molecules/mL plasma	19.9	1.56	2.08	2.13	0.37	0.59	0.65	0.84	0.35	0	0.74		0.23	0.42		
	MAF, %	1.70	0.40	0.45	0.27	0.05	0.14	0.05	0.09	0.02	0	0.04		0.03	0.09		
Mutation 2 NCOR1	Total number of molecules detected[a]	3485	1045	1007	1536	2108	1273	3375	3235	5256	955	3563		2095	1157		
	ctDNA, molecules/mL plasma	30.9	3.57	2.60	1.06	0	0	0	0	0	0.74	1.98		0	1.87		
	MAF, %	2.30	0.77	0.50	0.13	0	0	0	0	0	0.16	0.11		0	0.39		
	Total ctDNA, molecules/mL plasma	50.8	5.13	4.68	3.19	0.37	0.59	0.65	0.84	0.35	0.74	2.72		0.23	2.29		
	Imaging result				SD	SD	SD	SD	SD	SD	SD	SD	SD	SD	PD		

Patient 114		Pre-T	C1d15	C2d1	Scan 1
	Days from C1d1	0	15	29	70
	Plasma, mL	2.40	2.10	2.95	3.05
	Cell-free DNA, ng	21.0	29.9	18.5	26.5
	Amount of DNA analysed, ng	1.91	2.72	1.68	2.41
Mutation 1 *CBL*	Total number of molecules detected[a]	469	595	370	640
	ctDNA, molecules/mL plasma	16.7	0	0	16.4
	MAF, %	0.85	0	0	0.78
Mutation 2 *NOTCH3*	Total number of molecules detected[a]	117	111	100	95
	ctDNA, molecules/mL plasma	10.4	0	0	0
	MAF, %	2.00	0	0	0
Mutation 3 *FAT3*	Total number of molecules detected[a]	127	128	123	144
	ctDNA, molecules/mL plasma	0	0	0	3.28
	MAF, %	0	0	0	0.69
	Total ctDNA, molecules/mL plasma	27.1	0	0	19.7
	Imaging result				PD

Patient 115		Pre-T	C1d15	C2d1	Scan 1	Scan 2	Scan 3	Scan 4[b]	
	Days from C1d1	0	14	28	106	188	272	409	
	Plasma, mL	2.85	2.65	3.20	2.50	3.05	2.75	2.65	
	Cell-free DNA, ng	18.1	12.7	27.1	9.63	13.1	15.5	18.8	
	Amount of DNA analysed, ng	14.5	10.2	21.7	7.70	10.5	12.4	15.0	
Mutation 1 *CDH1*	Total number of molecules detected[a]	878	477	1039	396	622	855	976	
	ctDNA, molecules/mL plasma	17.3	0	0.20	0	0	0	75.2	
	MAF, %	4.50	0	0.05	0	0	0	16.4	
Mutation 2 *INPPL1*	Total number of molecules detected[a]	1802	997	2111	781	1209	1689	1715	
	ctDNA, molecules/mL plasma	38.6	0	0.78	0.50	0.20	0	132	
	MAF, %	4.88	0	0.09	0.13	0.04	0	16.3	
	Total ctDNA, molecules/mL plasma	55.80	0	0.98	0.50	0.20	0	207	
	Imaging result				SD	SD	SD	PD*	*SD on CT, but PD on MRI.

Patient 116		Pre-T	C1d15	C2d1	Scan 1	Scan 2	Scan 3	Scan 4	Scan 5	Scan 6	Scan 7	Scan 8
	Days from C1d1	0	15	29	70	154	238	323	406	490	574	688
	Plasma, mL	3.00	2.50	2.30	2.75	2.85	3.35	3.25	2.70	2.55	2.50	2.30
	Cell-free DNA, ng	63.8	16.8	23.3	20.0	19.9	19.1	13.5	12.4	8.09	9.90	7.81
	Amount of DNA analysed, ng	9.28	1.53	2.12	1.82	1.81	1.74	1.23	1.13	6.47	0.90	6.25

Mutation 1 *GATA3*	Total number of molecules detected[a]	475	115	237	148	200	183	131	197	478	176	521	
	ctDNA, molecules/mL plasma	125	12.0	0	0	0	0	0	0	0	0	0	
	MAF, %	11.0	2.60	0	0	0	0	0	0	0	0	0	
	Imaging result				PR	PR	PR	PR	PR	PR	PR	PD	
**Patient 118**		**Pre-T**	**C1d15**	**C2d1**	**Scan 1**	**Scan 2**	**Scan 3[b]**						
	Days from C1d1	0	16	31	72	156	238						
	Plasma, mL	3.35	2.50	2.60	2.80	2.75	3.50						
	Cell-free DNA, ng	139	27.9	40.2	26.5	36.1	787						
	Amount of DNA analysed, ng	20.2	2.54	3.66	2.41	3.29	40.0						
Mutation 1 *FAT2*	Total number of molecules detected[a]	2754	399	1079	621	472	5538						
	ctDNA, molecules/mL plasma	340	28.0	73.1	8.93	38.2	2135						
	MAF, %	6.00	1.80	1.76	0.40	2.20	6.90						
Mutation 2 *ESR1*	Total number of molecules detected[a]	298	67	115	62	71	544						
	ctDNA, molecules/mL plasma	29.8	0	0	5.36	5.45	213						
	MAF, %	4.90	0	0	2.44	2.13	7.00						
Mutation 3 PIK3CA	Total number of molecules detected[a]	859	97	277	156	95	2368						
	ctDNA, molecules/mL plasma	515	44.0	169	32.1	47.3	4298						
	MAF, %	29.0	11.0	16.0	5.77	13.8	32.3						
Mutation 4 *DOT1L*	Total number of molecules detected[a]	279	51	89	65	52	485						
	ctDNA, molecules/mL plasma	19.5	0	9.62	3.57	3.64	213						
	MAF, %	3.40	0	2.80	1.55	1.94	7.84						
Mutation 5 *CHD4*	Total number of molecules detected[a]	1007	87	349	206	101	2148						
	ctDNA, molecules/mL plasma	184	0	32.7	26.8	5.45	1177						
	MAF, %	7.40	0	1.40	3.65	1.49	9.76						
	Total ctDNA, molecules/mL plasma	1088	72.0	284	76.8	100	8036						
	Imaging result				SD	PR	PD						
**Patient 119**		**Pre-T**	**C1d15**	**C2d1**	**Scan 1**	**Scan 2**	**Scan 3**	**Scan 4**	**Scan 5**	**Scan 6**	**Scan 7**	**Scan 8**	**Scan 9[b]**
	Days from C1d1	0	16	30	71	155	243	322	400	490	574	658	778
	Plasma, mL	3.10	2.50	2.70	3.05	3.20	3.40	3.40	2.15	3.00	3.00	2.20	3.10
	Cell-free DNA, ng	842	135	48.8	18.9	16.6	22.4	16.1	7.87	12.9	24.1	8.91	23.4
	Amount of DNA analysed, ng	40.0	19.6	4.44	1.72	1.51	2.04	1.46	6.29	10.3	2.19	7.13	18.7
Mutation 1 *SF3B1*	Total number of molecules detected[a]	3545	1036	450	195	244	244	207	456	809	205	624	1707
	ctDNA, molecules/mL plasma	10889	521	88.9	0	0	0	0	0	0	0	1.99	35.5
	MAF, %	45.3	18.3	5.33	0	0	0	0	0	0	0	0.56	5.16

Mutation 2 *TP53*	Total number of molecules detected[a]	2643	1340	604	304	426	468	356	913	1426	343	1049	2590
	ctDNA, molecules/mL plasma	14370	813	131	0	0	0	0	0	0	0	3.41	44.0
	MAF, %	80.1	22.1	5.88	0	0	0	0	0	0	0	0.57	4.21
	Total ctDNA, molecules/mL plasma	25259	1334	220	0	0	0	0	0	0	0	5.40	79.4
	Imaging result				SD	PR	PR	PR	PR	PR	PR	PR	PD
**Patient 121**		**Pre-T**	**C1d15**	**C2d1**	**Scan 1**	**Scan 2**	**Scan 3**	**Scan 4**	**Scan 5**	**Scan 6**	**Scan 7**	**Scan 8**	**Scan 9**
	Days from C1d1	0	13	28	70	154	250	325	411	521	672	805	924
	Plasma, mL	2.80	2.50	2.45	1.95	3.30	2.70	2.60	2.30	3.15	1.80	2.00	3.50
	Cell-free DNA, ng	38.6	34.9	90.8	9.08	11.3	5.56	6.93	5.28	10.9	17.1	20.7	35.4
	Amount of DNA analysed, ng	3.51	3.17	13.2	0.83	1.03	4.44	5.54	4.22	8.76	13.0	16.7	28.2
No mutations detected	ctDNA, molecules/mL plasma	0	0	0	0	0	0	0	0	0	0	0	0
	Imaging result				SD	PR	PR	PR	PR	PR	PR	PR	PR
**Patient 123**		**Pre-T**	**C1d15**	**C2d1**	**Scan 1[b]**	.							
	Days from C1d1	0	15	29	168								
	Plasma, mL	3.20	2.15	3.00	2.70								
	Cell-free DNA, ng	241	69.9	96.3	255								
	Amount of DNA analysed, ng	35.0	10.2	14.0	18.6								
Mutation 1 *PIK3CA_1*	Total number of molecules detected[a]	103	17	9	611								
	ctDNA, molecules/mL plasma	46.2	0	6.88	912								
	MAF, %	20.9	0	33.3	29.3								
Mutation 2 *PIK3CA_2*	Total number of molecules detected[a]	1705	425	542	1710								
	ctDNA, molecules/mL plasma	1183	166	245	2663								
	MAF, %	32.3	12.2	19.8	30.6								
Mutation 3 *TP53_1*	Total number of molecules detected[a]	1468	379	472	1811								
	ctDNA, molecules/mL plasma	967	138	233	2462								
	MAF, %	30.7	11.3	21.5	26.7								
Mutation 4 *TP53_2*	Total number of molecules detected[a]	1284	282	438	1124								
	ctDNA, molecules/mL plasma	1183	163	308	1902								
	MAF, %	42.9	18.1	30.7	33.2								
	Total ctDNA, molecules/mL plasma	3379	467	793	7939								
	Imaging result				PD								

Patient 124		Pre-T	C1d15	C2d1	Scan 1	Scan 2	Scan 3	Scan 4	Scan 5	Scan 6	Scan 7	
	Days from C1d1	0	15	29	70	154	245	322	406	518	630	
	Plasma, mL	3.10	2.15	3.30	2.52	2.65	3.10	2.35	2.20	2.45	2.50	
	Cell-free DNA, ng	12.5	8.25	28.8	6.11	8.86	9.85	9.24	8.86	7.48	22.2	
	Amount of DNA analysed, ng	10.0	6.60	23.0	4.88	7.08	7.88	7.39	7.08	5.98	17.8	
No mutations detected	ctDNA (molecules/mL plasma), n	0	0	0	0	0	0	0	0	0	0	
	Imaging result				SD	SD	SD	SD	SD	SD	SD	
**Patient 125**		**Pre-T**	**C1d15**	**C2d1**	**Scan 1**	**Scan 2**	**Scan 3**	**Scan 4**	**Scan 5**	**Scan 6**	**Scan 7**	
	Days from C1d1	0	15	29	70	161	273	350	483	588	700	
	Plasma, mL	3.30	3.30	3.50	2.05	3.00	2.40	3.1	3.13	3.50	4.00	
	Cell-free DNA, ng	17.7	22.7	20.5	6.27	7.43	11.8	12.8	9.63	22.1	38.1	
	Amount of DNA analysed, ng	14.1	18.2	16.4	5.02	5.94	9.42	10.2	7.70	17.6	30.5	
Mutation 1 *ERBB3*	Total number of molecules detected[a]	822	1085	1047	210	317	539	485	344	745	1261	
	ctDNA, molecules/mL plasma	1.33	0	0	0	0	0	0	0	0	0	
	MAF, %	0.42	0	0	0	0	0	0	0	0	0	
Mutation 2 *SPOP*	Total number of molecules detected[a]	1308	1638	1503	279	480	810	670	481	1144	2160	
	ctDNA, molecules/mL plasma	1.70	0	0.18	0	0	0	0	0	0	0	
	MAF, %	0.34	0	0.03	0	0	0	0	0	0	0	
Mutation 3 *TP53*	Total number of molecules detected[a]	500	614	530	199	265	252	502	333	791	1441	
	ctDNA, molecules/mL plasma	0.38	0	0	0	0	0	0	0	0	0	
	MAF, %	0.20	0	0	0	0	0	0	0	0	0	
Mutation 4 *MAP2K*	Total number of molecules detected[a]	323	339	302	164	211	156	495	344	857	2512	
	ctDNA, molecules/mL plasma	0.38	0	0	0	0	0	0.20	0	0	0	
	MAF, %	0.31	0	0	0	0	0	0.10	0	0	0	
	Total ctDNA, molecules/mL plasma	3.76	0	0.17	0	0	0	0.20	0	0	0	
	Imaging result				SD	SD	SD	SD	SD	SD	SD	
**Patient 126**		**Pre-T**	**C1d15**	**C2d1**	**Scan 1**	**Scan 2**	**Scan 3**	**Scan 4**	**Scan 5**	**Scan 6**	**Scan 7**	**Scan 8**
	Days from C1d1	0	15	29	70	154	236	322	406	518	630	714
	Plasma, mL	2.70	2.00	2.80		2.20	2.55	2.05	2.70	2.75	2.80	4.00
	Cell-free DNA, ng	30.3	7.10	15.7	Missing	14.1	11.2	8.31	12.3	9.35	32.9	41.4
	Amount of DNA analysed, ng	24.2	5.68	12.5		11.3	8.93	8.93	11.3	7.48	26.3	33.1
Mutation 1 *MAP3K1*	Total number of molecules detected[a]	343	98	121		613	83	285	454	296	801	916
	ctDNA, molecules/mL plasma	0.23	0	0		0	0	0	0	0	0	0
	MAF, %	0.15	0	0		0	0	0	0	0	0	0
	Imaging result				SD	SD	SD	SD	SD	SD	SD	SD

Patient 201		Pre-T	C1d15	C2d1	Scan 1	Scan 2	Scan 3	Scan 4	Scan 5	Scan 6	Scan 7	Scan 8
	Days from C1d1	0	15	29	76	159	257	348	478	621	749	868
	Plasma, mL	2.50	2.40	2.80	2.20	3.00	3.00	2.30	3.00	2.80	3.20	3.08
	Cell-free DNA, ng	5.72	2.75	6.38	7.10	11.7	10.2	7.21	19.6	9.19	17.8	9.30
	Amount of DNA analysed, ng	4.16	2.00	4.64	2.58	8.48	7.44	5.24	15.7	7.35	14.3	7.44
No mutations detected	ctDNA (molecules/mL plasma), n	0	0	0	0	0	0	0	0	0	0	0
	Imaging result				SD	PR	PR	PR	PR	PR	PR	PR
**Patient 202**		**Pre-T**	**C1d15**	**C2d1**	**Scan 1**	**Scan 2[b]**	**Scan 3[b]**	**Scan 4**				
	Days from C1d1	0	14	27	71	153	244	352				
	Plasma, mL	2.00	2.85	2.50	3.10	2.50	2.80	2.95				
	Cell-free DNA, ng	450	80.9	94.6	26.3	49.7	31.5	93.5				
	Amount of DNA analysed, ng	20.0	11.8	13.8	19.2	36.2	22.9	13.6				
Mutation 1 *BRCA2*	Total number of molecules detected[a]	1461	217	290	125	372	79	38				
	ctDNA, molecules/mL plasma	2889	47.0	129	1.01	0.50	6.47	25.6				
	MAF, %	17.6	9.00	16.2	2.01	0.27	18.5	28.9				
	Imaging result				SD	SD	PR	PD				
**Patient 203**		**Pre-T**	**C1d15**	**C2d1**	**Scan 1**							
	Days from C1d1	0	19	28	74							
	Plasma, mL	2.80	1.30	2.30	2.50							
	Cell-free DNA, ng	99.0	10.5	21.7	19.7							
	Amount of DNA analysed, ng	14.4	7.64	15.8	14.3							
Mutation 1 *CDH1*	Total number of molecules detected[a]	850	689	2049	1322							
	ctDNA, molecules/mL plasma	982	19.2	9.78	0							
	MAF, %	23.5	1.45	0.44	0							
Mutation 2 *TP53*	Total number of molecules detected[a]	1554	2235	4586	3667							
	ctDNA, molecules/mL plasma	830	175	60.6	42.5							
	MAF, %	21.8	8.17	2.43	2.32							
Mutation 3 *SNORD9*	Total number of molecules detected[a]	1476	1617	3460	2831							
	ctDNA, molecules/mL plasma	559	22.6	7.88	0.50							
	MAF, %	15.4	1.45	0.42	0.04							
Mutation 4 *FGFR4*	Total number of molecules detected[a]	2457	2526	5557	4289							
	ctDNA, molecules/mL plasma	422	43.3	16.3	0							
	MAF, %	7.00	1.78	0.54	0							

Mutation 5 *FGF19*	Total number of molecules detected[a]	2216	2434	4552	3612	
	ctDNA, molecules/mL plasma	438	46.6	19.3	0	
	MAF, %	8.06	1.99	0.78	0	
	Total ctDNA, molecules/mL plasma	3231	307	114	43	
	Imaging result				SD	
**Patient 205**		**Pre-T**	**C1d15**	**C2d1**	**Scan 1**	
	Days from C1d1	0	15	28	42	
	Plasma, mL	2.60	3.10	3.00	2.60	
	Cell-free DNA, ng	19.5	21.5	15.9	144	
	Amount of DNA analysed, ng	14.2	15.6	11.6	20.9	
Mutation 1 *SOX9*	Total number of molecules detected[a]	2106	2164	1637	1706	
	ctDNA, molecules/mL plasma	62.7	48.4	90.8	1027	
	MAF, %	6.20	5.55	13.3	22.8	
Mutation 2 *CDK8*	Total number of molecules detected[a]	21	0	0	14	
	ctDNA, molecules/mL plasma	0.96	0	0	6.61	
	MAF, %	9.52	0	0	18.5	
Mutation 3 *MYH9*	Total number of molecules detected[a]	1186	1101	915	891	
	ctDNA, molecules/mL plasma	41.8	25.8	47.5	508	
	MAF, %	7.34	5.82	12.5	21.5	
Mutation 4 *PIK3CA*	Total number of molecules detected[a]	265	144	189	128	
	ctDNA, molecules/mL plasma	11.1	3.23	11.9	75.4	
	MAF, %	8.68	5.56	15.1	22.3	
Mutation 5 *FGFR4_1*	Total number of molecules detected[a]	6330	5280	5417	5029	
	ctDNA, molecules/mL plasma	248	141	323	2786	
	MAF, %	8.14	6.64	14.3	21.0	
Mutation 6 *NF1*	Total number of molecules detected[a]	1232	1198	814	1069	
	ctDNA, molecules/mL plasma	50.2	26.6	54.4	740	
	MAF, %	8.48	5.51	16.0	26.2	
Mutation 7 *FGFR4_2*	Total number of molecules detected[a]	3015	2971	2546	2904	
	ctDNA, molecules/mL plasma	101	64.3	144	1212	
	MAF, %	6.97	5.37	13.6	15.8	
	Total ctDNA, molecules/mL plasma	516	309	672	6355	
	Imaging result				PD*	*Clinial PD confirmed by imaging.

Patient 206		Pre-T	C1d15	C2d1	Scan 1[b]	Scan 2	Scan 3	Scan 4	Scan 5	Scan 6
	Days from C1d1	0	16	28	84	164	303	440	560	674
	Plasma, mL	2.80	2.00	3.10	2.40	2.30	2.80	2.75	2.17	2.85
	Cell-free DNA, ng	23.4	11.0	18.9	10.6	12.0	23.0	24.0	10.8	18.0
	Amount of DNA analysed, ng	17.0	8.00	13.7	7.68	8.72	18.4	19.2	8.62	14.4
Mutation 1 *PIK3CA_1*	Total number of molecules detected[a]	1249	651	744	485	487	311	381	155	341
	ctDNA, molecules/mL plasma	30.8	0	1.41	0.52	0	0	0	0	0
	MAF, %	5.52	0	0.47	0.21	0	0	0	0	0
Mutation 2 *PTPRC*	Total number of molecules detected[a]	2160	1056	1196	722	785	925	938	85	286
	ctDNA, molecules/mL plasma	53.6	0.94	1.01	0	0	0	0	0	0
	MAF, %	5.56	0.14	0.21	0	0	0	0	0	0
Mutation 3 *SMARCA4*	Total number of molecules detected[a]	2126	940	1171	638	767	49	78	272	650
	ctDNA, molecules/mL plasma	80.8	0.94	2.02	0.52	0.54	0	0	0	0
	MAF, %	8.52	0.16	0.43	0.16	0.13	0	0	0	0
Mutation 4 *FAT2*	Total number of molecules detected[a]	5318	2596	3537	2131	2285	2650	2637	394	1089
	ctDNA, molecules/mL plasma	152	5.00	5.04	3.39	0	0	0	0	0
	MAF, %	6.38	0.31	0.35	0.31	0	0	0	0	0
Mutation 5 *CDH1*	Total number of molecules detected[a]	1580	811	785	397	711	49	82	356	859
	ctDNA, molecules/mL plasma	15.4	1.25	0.40	0	0	0	0	0	0
	MAF, %	2.18	0.25	0.13	0	0	0	0	0	0
Mutation 6 *PIK3CA_2*	Total number of molecules detected[a]	4111	1873	2386	1447	1602	1312	1443	215	567
	ctDNA, molecules/mL plasma	339	3.13	9.27	2.86	1.36	0	0.68	0	1.10
	MAF, %	18.5	0.27	0.96	0.38	0.16	0	0.10	0	0.44
	Total ctDNA, molecules/mL plasma	672	11.3	19.2	7.29	1.9	0	0.68	0	1.10
	Imaging result				SD	SD	SD	SD	SD	SD

Patient 207		Pre-T	C1d15	C2d1	Scan 1	Scan 2	Scan 3[b]
	Days from C1d1	0	14	28	67	157	231
	Plasma, mL	2.60	2.60	2.60	2.40	2.80	2.10
	Cell-free DNA, ng	20.7	9.13	23.0	24.6	88.6	290
	Amount of DNA analysed, ng	15.0	6.64	17.7	17.9	6.44	21.1
Mutation 1 *INPPL1*	Total number of molecules detected[a]	2478	1761	2636	2187	741	2262
	ctDNA, molecules/mL plasma	90.1	42.3	77.6	38.0	1181	2354
	MAF, %	7.57	5.00	6.49	3.34	32.5	15.9
Mutation 2 *CDH1*	Total number of molecules detected[a]	2583	1909	2606	2359	716	2718
	ctDNA, molecules/mL plasma	59.9	31.0	60.3	27.6	879	2007
	MAF, %	4.82	3.38	5.10	2.25	25.0	11.3

Mutation 3 *TBX3*	Total number of molecules detected[a]	9	3	75	19	9	28				
	ctDNA, molecules/mL plasma	0.72	0.48	6.12	1.56	24.6	75.3				
	MAF, %	17.6	33.3	18.1	16.2	55.6	41.1				
Mutation 4 *H3-5*	Total number of molecules detected[a]	34	15	176	77	32	107				
	ctDNA, molecules/mL plasma	0.48	0	2.50	0	14.7	49.1				
	MAF, %	2.99	0	3.13	0	9.38	7.01				
	Total ctDNA, molecules/mL plasma	151	73.8	147	67.2	2099	4485				
	Imaging result				SD	SD	PD				
**Patient 208**		**Pre-T**	**C1d15**	**C2d1**	**Scan 1**	**Scan 2**	**Scan 3**	**Scan 4**	**Scan 5**	**Scan 6**	**Scan 7**
	Days from C1d1	0	15	28	70	154	244	321	406	518	609
	Plasma, mL	1.90	2.90	2.70	2.90	3.00	1.60	3.55	3.15	3.00	2.97
	Cell-free DNA, ng	28.1	11.7	21.4	14.3	20.5	14.3	26.5	17.8	16.3	21.9
	Amount of DNA analysed, ng	22.5	9.37	17.1	11.4	16.4	11.4	21.2	14.2	13.0	17.6
Mutation 1 *AKT1*	Total number of molecules detected[a]	204	70	78	38	103	19	136	1138	871	1194
	ctDNA, molecules/mL plasma	2.63	0	0	0	0	0	0	0	0	0
	MAF, %	1.97	0	0	0	0	0	0	0	0	0
Mutation 2 *RUNX1*	Total number of molecules detected[a]	61	20	17	16	37	4	31	728	568	956
	ctDNA, molecules/mL plasma	0.99	0	0	0	0	0	0	0	0	0
	MAF, %	2.46	0	0	0	0	0	0	0	0	0
	Total ctDNA, molecules/mL plasma	3.61	0	0	0	0	0	0	0	0	0
	Imaging result				PR	PR	PR	PR	PR	PR	PR
**Patient 213**		**Pre-T**	**C1d15**	**C2d1**	**Scan 1**	**Scan 2**	**Scan 3**	**Scan 4**			
	Days from C1d1	0	19	28	74	182	278	376			
	Plasma, mL	2.00	2.95	3.20	3.25	2.20	2.97	3.15			
	Cell-free DNA, ng	15.5	18.6	19.1	34.0	7.21	20.6	15.1			
	Amount of DNA analysed, ng	12.4	14.9	15.3	27.2	5.76	16.5	12.1			
No mutations detected	ctDNA, molecules/mL plasma	0	0	0	0	0	0	0			
	Imaging result				SD	SD	SD	SD			

Abbreviations: C1d15, cycle 1 day 15, C2d1, cycle 2 day 1; ctDNA, circulating tumour DNA; CT, computed tomography; CDK4/6i, cycline dependent kinase 4/6 inhibitor; MAF, mutant allele frequency; MRI, magnetic resonance imaging, PD, progressive disease; PR, partial response; pre-T, pre-treatment; RECIST, Response Evaluation Criteria In Solid Tumours; SD, stable disease.

[a] Including wildtype and ctDNA molecules

[b] Blood samples collected off CDK4/6i

**Supplementary Table 4A. Original and altered criteria and limit function used to define ctDNA rise.**			
	**Original criteria**		
(1)	reappearance of ctDNA if $ctDNA_{nadir}$ was below the threshold for detection **or** detection of ctDNA if previously undetected	if 2 reads för SNV, 1 read för InDel, **and** MAF >0.1 %	$ctDNA_{nadir} \times (1.5 + 1.47 \times e^{-0.39 \times ctDNAnadir})$
(2)	>3-fold increase in ctDNA level if $ctDNA_{nadir}$ ≤ 1 molecule/mL		
(3)	>2-fold increase if $ctDNA_{nadir}$ >1–5 molecules/mL		
(4)	>1.5-fold increase if $ctDNA_{nadir}$ >5 molecules/mL		
	**Lower sensitivity**		
(1)	reappearance of ctDNA if $ctDNA_{nadir}$ was below the threshold for detection **or** detection of ctDNA if previously undetected	if 3 reads för SNV, 1 read för InDel, **and** MAF >0.1 %	$ctDNA_{nadir} \times (1.75 + 2.20 \times e^{-0.39 \times ctDNAnadir})$
(2)	>4-fold increase in ctDNA level if $ctDNA_{nadir}$ ≤ 1 molecule/mL		
(3)	>2.5-fold increase if $ctDNA_{nadir}$ >1–5 molecules/mL		
(4)	>1.75-fold increase if $ctDNA_{nadir}$ >5 molecules/mL		
	**Higher sensitivity**		
(1)	reappearance of ctDNA if $ctDNA_{nadir}$ was below the threshold for detection **or** detection of ctDNA if previously undetected	if 2 reads för SNV, 1 read för InDel, **and** MAF >0.1 %	$ctDNA_{nadir} \times (1.25 + 0.73 \times e^{-0.39 \times ctDNAnadir})$
(2)	>4-fold increase in ctDNA level if $ctDNA_{nadir}$ ≤ 1 molecule/mL		
(3)	>2.5-fold increase if $ctDNA_{nadir}$ >1–5 molecules/mL		
(4)	>1.75-fold increase if $ctDNA_{nadir}$ >5 molecules/mL		

Supplementary Table 4B. Time point for ctDNA rise using original and altered criteria.

Patient	Original criteria	Lower sensitivity	Higher sensitivity	PD time point
101	No ctDNA rise	No ctDNA rise	No ctDNA rise	No PD
104	Scan 3	Scan 3	Scan 3	Scan 5
107	Scan 7	**Scan 8**	Scan 7	Scan 9
109	No ctDNA rise	No ctDNA rise	No ctDNA rise	No PD
110	Scan 4	Scan 4	**Scan 3**	Scan 4
113	Scan 7	Scan 7	Scan 7	Scan 11
114	Scan 1	Scan 1	Scan 1	Scan 1
115	Scan 4	Scan 4	Scan 4	Scan 4
116	No ctDNA rise	No ctDNA rise	No ctDNA rise	Scan 8
118	Scan 1	Scan 1	Scan 1	Scan 3
119	Scan 8	Scan 8	Scan 8	Scan 9
123	Scan 2	Scan 2	Scan 2	Scan 2
125	Scan 4	Scan 4	Scan 4	No PD
126	No ctDNA rise	No ctDNA rise	No ctDNA rise	No PD
202	Scan 3	Scan 3	Scan 3	Scan 4
203	No ctDNA rise	No ctDNA rise	No ctDNA rise	No PD
205	Scan 1	Scan 1	**C2d1**	Scan 1
206	Scan 4	Scan 4	Scan 4	No PD
207	C2d1	C2d1	C2d1	Scan 1
208	No ctDNA rise	No ctDNA rise	No ctDNA rise	No PD
213	ctDNA ND	ctDNA ND	ctDNA ND	No PD
124	ctDNA ND	ctDNA ND	ctDNA ND	No PD
201	ctDNA ND	ctDNA ND	ctDNA ND	No PD
121	ctDNA ND	ctDNA ND	ctDNA ND	No PD

Supplementary Table 4C. Outcomes using original and altered critiera for ctDNA rise.

	Original criteria	Lower sensitivity	Higher sensitivity
ctDNA rise, n	14	14	14
ctDNA rise prior to PD	7	7	9
ctDNA rise at PD	5	5	3
ctDNA rise without PD	2	2	2
PD without ctDNA rise	1	1	1
Median/mean lead-time ctDNA rise to PD, days (min-max)	114/120 (0-450)	110/110 (0-450)	114/128 (0-450)
Number of 'unnecessary'scans	71	70	69

Abbreviations: ctDNA, circulating tumour DNA; ND, not detected; MAF, mutant allele frequency; PD, progressive disease; SNV, single nucleotide variant.

Supplementary Figures

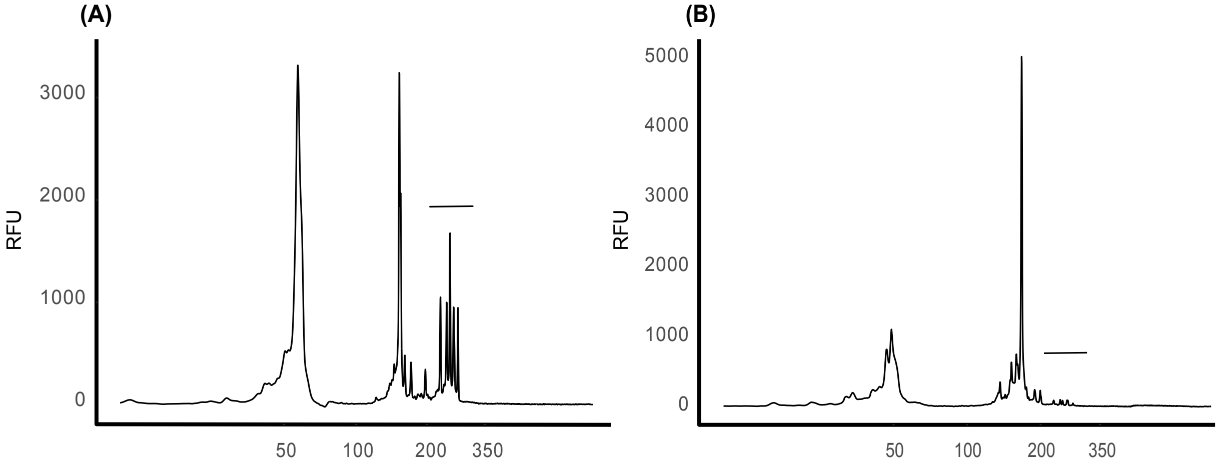

Supplementary Figure 1. Evaluation of ctDNA panels based on fragment analysis. Horizontal lines indicate target libraries of 210-300 base pairs. (A) An example of a ctDNA panel used for plasma ctDNA analysis (patient 101) that meets the criteria of ≥ 10 nmol/L target library product (horizontal lines). (B) An example of a ctDNA panel that does not meet the criteria and therefore needs to be repurchased or redesigned (patient not included in the study). *Abbreviations: ctDNA, circulating tumour DNA; RFU, relative fluorescence unit.*

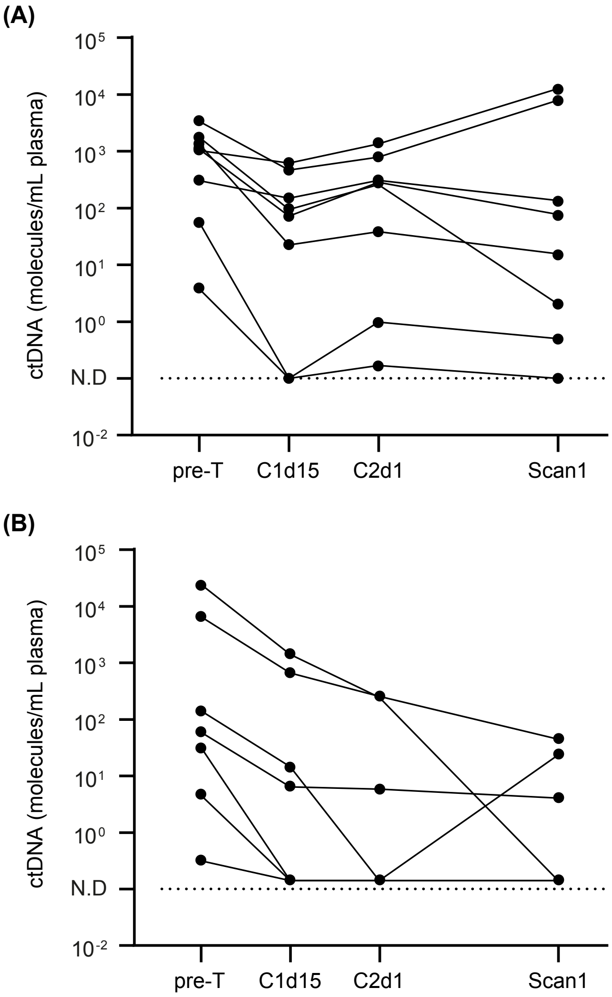

Supplementary Figure 2. Early ctDNA dynamics for patients with available samples at the first three time points. (A) Patients with a rise in total ctDNA level from C1d15 to C2d1 (n=8). (B) Patients with continued or further suppression of total ctDNA level from C1d15 to C2d1 (n=7).
Abbreviations: C1d15, cycle 1 day 15; C2d1, cycle 2 day 1; ctDNA, circulating tumour DNA; N.D, not detected; pre-T, pre-treatment.

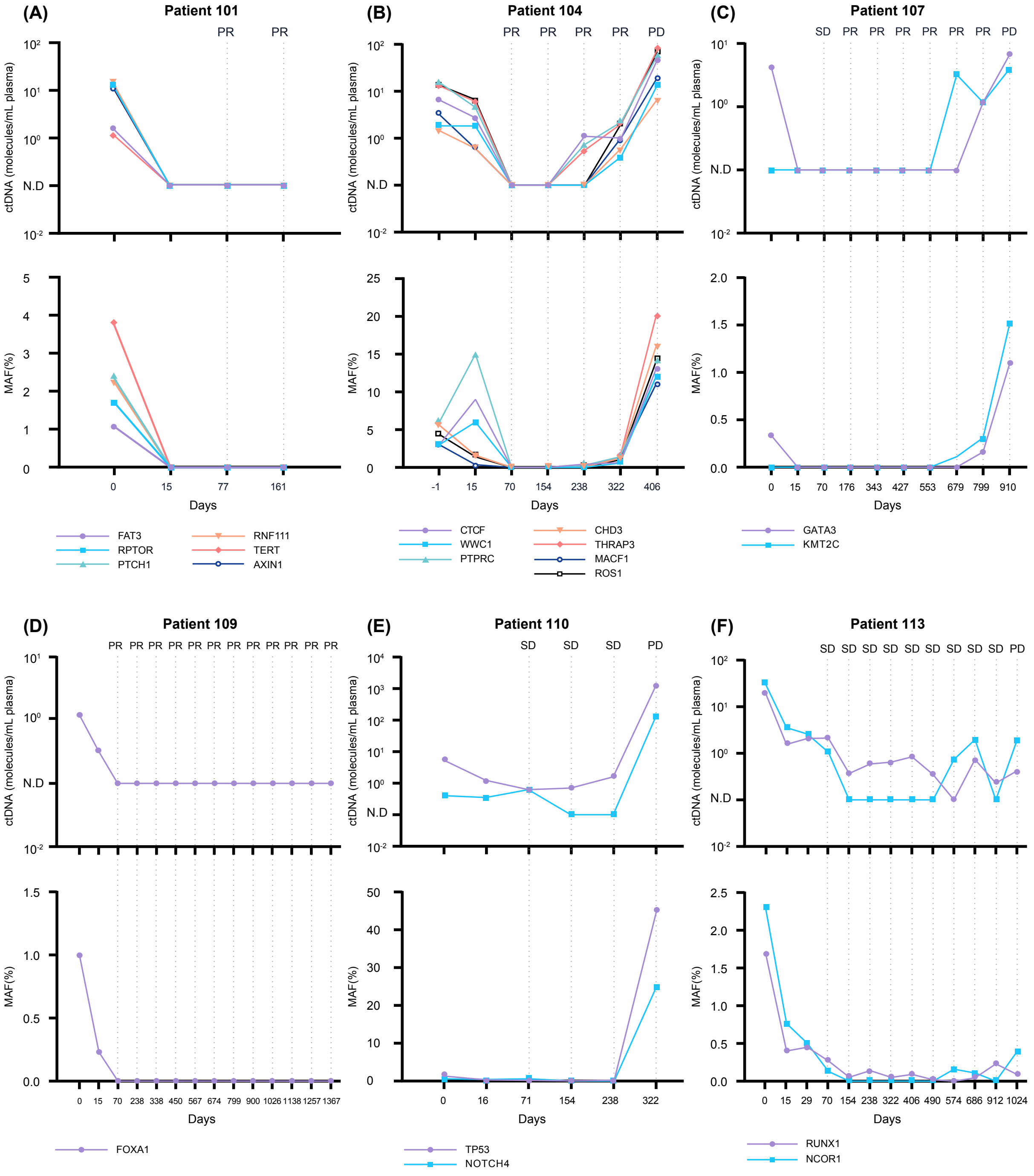

Supplementary Figure 3. Circulating tumour DNA levels and imaging results for each of the patients with detectable ctDNA (A to F). The top figure illustrates ctDNA levels reported as copies/mL plasma (log scale) and the bottom figure illustrates ctDNA levels reported as MAF.
Abbreviations: ctDNA, circulating tumour DNA; MAF, mutant allele frequency.

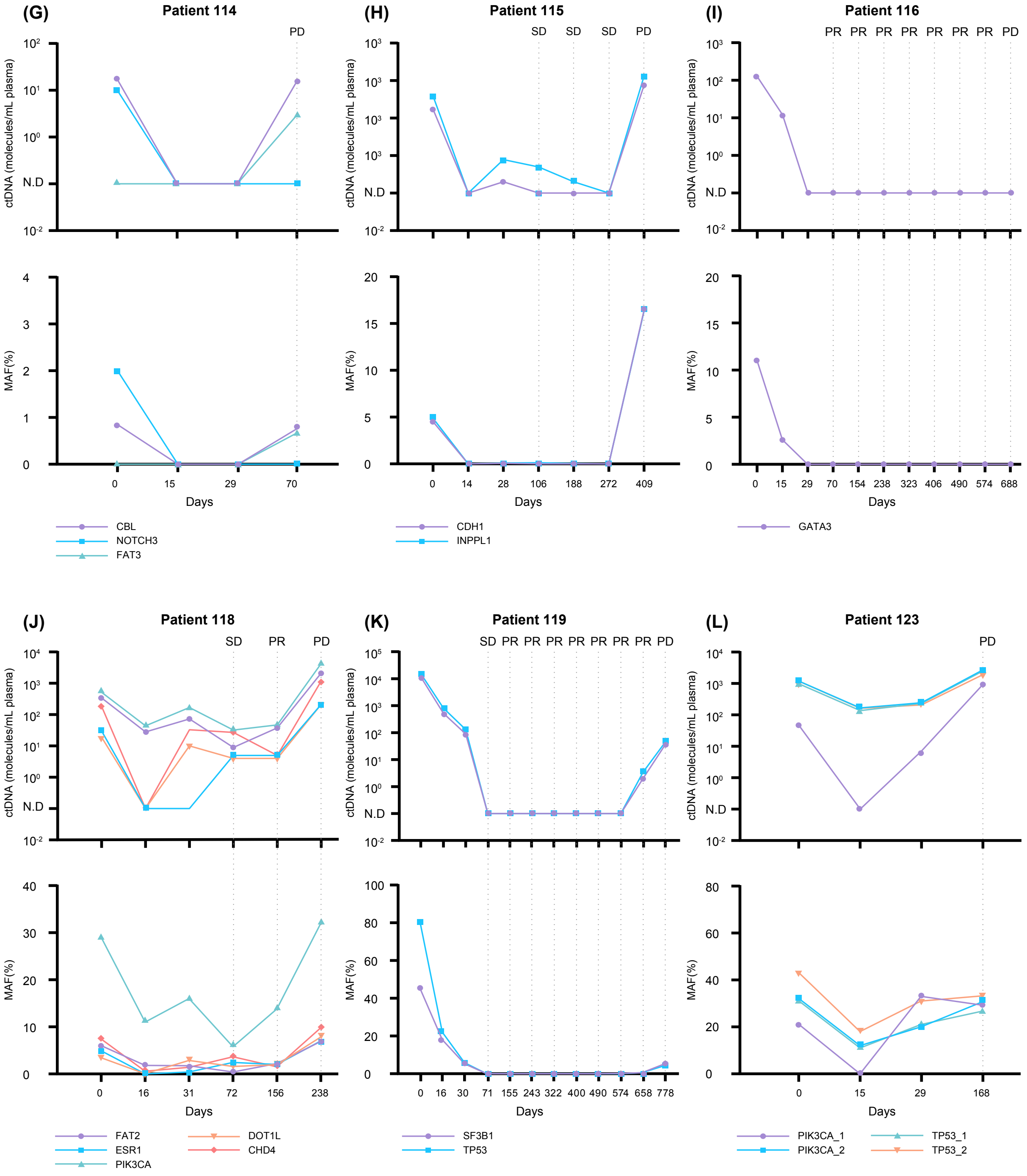

Supplementary Figure 3, continued. Circulating tumour DNA levels and imaging results for each of the patients with detectable ctDNA (G to L). The top figure illustrates ctDNA levels reported as copies/mL plasma (log scale) and the bottom figure illustrates ctDNA levels reported as MAF.
Abbreviations: ctDNA, circulating tumour DNA; MAF, mutant allele frequency.

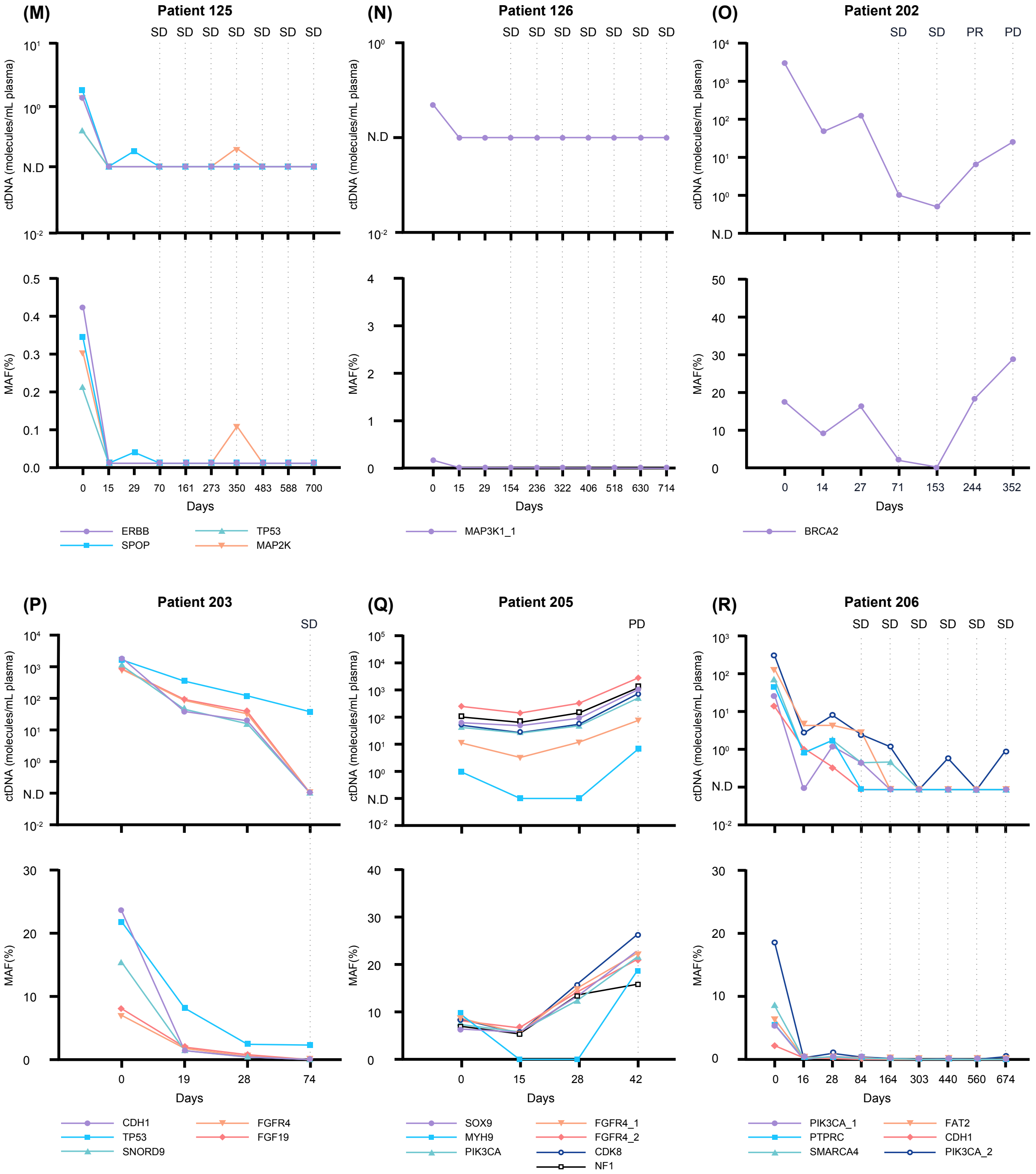

Supplementary Figure 3, continued. Circulating tumour DNA levels and imaging results for each of the patients with detectable ctDNA (M to R). The top figure illustrates ctDNA levels reported as copies/mL plasma (log scale) and the bottom figure illustrates ctDNA levels reported as MAF.
Abbreviations: ctDNA, circulating tumour DNA; MAF, mutant allele frequency.

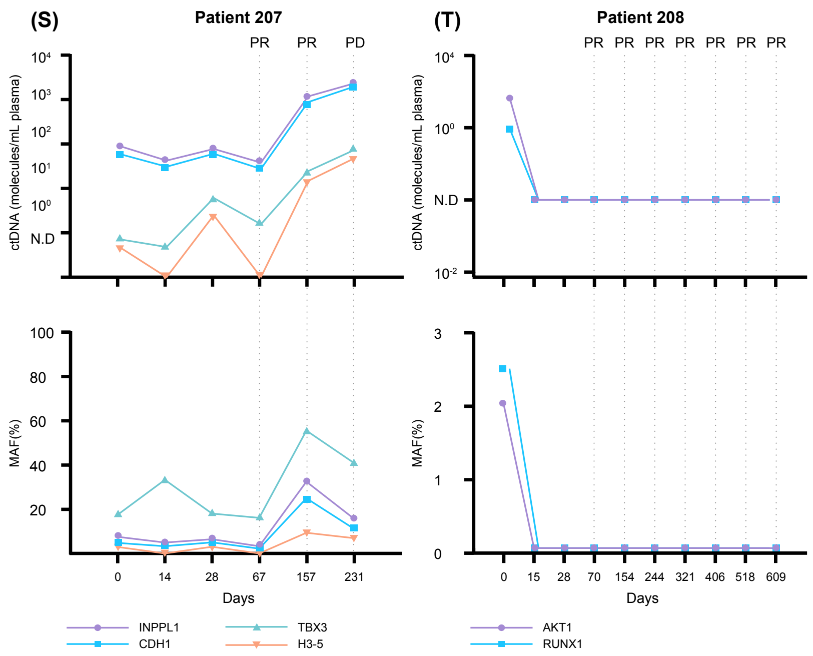

Supplementary Figure 3, continued. Circulating tumour DNA levels and imaging results for each of the patients with detectable ctDNA (S to T). The top figure illustrates ctDNA levels reported as copies/mL plasma (log scale) and the bottom figure illustrates ctDNA levels reported as MAF.
Abbreviations: ctDNA, circulating tumour DNA; MAF, mutant allele frequency.

Supplementary reference list

1. Eisenhauer EA, Therasse P, Bogaerts J, Schwartz LH, Sargent D, Ford R, Dancey J, Arbuck S, Gwyther S, Mooney M, Rubinstein L, Shankar L, et al. New response evaluation criteria in solid tumours: revised RECIST guideline (version 1.1). *European journal of cancer (Oxford, England : 1990)* 2009;**45**: 228-47.
2. Genomic Medicine Sweden. GMS560: a broad targeted NGS gene panel for comprehensive genomic profiling of solid tumors. 2023. https://genomicmedicine.se/wp-content/uploads/2023/11/GMS560_synopsis.pdf. Accessed January 2024.
3. Stahlberg A, Krzyzanowski PM, Egyud M, Filges S, Stein L, Godfrey TE. Simple multiplexed PCR-based barcoding of DNA for ultrasensitive mutation detection by next-generation sequencing. *Nat Protoc* 2017;**12**: 664-82.
4. Hu Y, Alden RS, Odegaard JI, Fairclough SR, Chen R, Heng J, Feeney N, Nagy RJ, Shah J, Ulrich B, Gutierrez M, Lanman RB, et al. Discrimination of Germline EGFR T790M Mutations in Plasma Cell-Free DNA Allows Study of Prevalence Across 31,414 Cancer Patients. *Clinical cancer research : an official journal of the American Association for Cancer Research* 2017;**23**: 7351-9.
5. Osterlund T, Filges S, Johansson G, Stahlberg A. UMIErrorCorrect and UMIAnalyzer: Software for Consensus Read Generation, Error Correction, and Visualization Using Unique Molecular Identifiers. *Clin Chem* 2022;**68**: 1425-35.
6. Andersson D, Fagman H, Dalin MG, Stahlberg A. Circulating cell-free tumor DNA analysis in pediatric cancers. *Mol Aspects Med* 2020;**72**: 100819.
7. Bos MK, Nasserinejad K, Jansen M, Angus L, Atmodimedjo PN, de Jonge E, Dinjens WNM, van Schaik RHN, Del Re M, Dubbink HJ, Sleijfer S, Martens JWM. Comparison of variant allele frequency and number of mutant molecules as units of measurement for circulating tumor DNA. *Mol Oncol* 2021;**15**: 57-66.
8. Oken MM, Creech RH, Tormey DC, Horton J, Davis TE, McFadden ET, Carbone PP. Toxicity and response criteria of the Eastern Cooperative Oncology Group. *American journal of clinical oncology* 1982;**5**: 649-55.
9. Union for International Cancer Control. *TNM classification of malignant tumours (8th ed.)*: Wiley-Blackwell, 2017.
